# The nuclear and mitochondrial genome assemblies of *Tetragonisca angustula* (Apidae: Meliponini), a tiny yet remarkable pollinator in the Neotropics

**DOI:** 10.1186/s12864-024-10502-z

**Published:** 2024-06-11

**Authors:** Rafael Rodrigues Ferrari, Paulo Cseri Ricardo, Felipe Cordeiro Dias, Natalia de Souza Araujo, Dalliane Oliveira Soares, Qing-Song Zhou, Chao-Dong Zhu, Luiz Lehmann Coutinho, Maria Cristina Arias, Thiago Mafra Batista

**Affiliations:** 1 Centro de Formação em Ciências Ambientais, Universidade Federal do Sul da Bahia, Porto Seguro, Brazil; 2 Key Laboratory of Zoological Systematics and Evolution, Institute of Zoology, Chinese Academy of Sciences, Beijing, China; 3 Departamento de Genética e Biologia Evolutiva, Instituto de Biociências, Universidade de São Paulo, São Paulo, Brazil; 4 Evolutionary Biology & Ecology, Université Libre de Bruxelles, Bruxelles, Belgium; 5 College of Life Sciences, University of Chinese Academy of Sciences, Beijing, China; 6 Sate Key Laboratory of Integrated Pest Management, Institute of Zoology, Chinese Academy of Sciences, Beijing, China; 7 Departamento de Ciências Animais, Universidade de São Paulo/ESALQ, Piracicaba, Brazil

**Keywords:** Functional annotation, Gene family, Genome assembly, Phylogeny, Transcriptome

## Abstract

**Background:**

The field of bee genomics has considerably advanced in recent years, however, the most diverse group of honey producers on the planet, the stingless bees, are still largely neglected. In fact, only eleven of the ~ 600 described stingless bee species have been sequenced, and only three using a long-read (LR) sequencing technology. Here, we sequenced the nuclear and mitochondrial genomes of the most common, widespread and broadly reared stingless bee in Brazil and other neotropical countries—*Tetragonisca angustula* (popularly known in Brazil as jataí).

**Results:**

A total of 48.01 Gb of DNA data were generated, including 2.31 Gb of Pacific Bioscience HiFi reads and 45.70 Gb of Illumina short reads (SRs). Our preferred assembly comprised 683 contigs encompassing 284.49 Mb, 62.84 Mb of which (22.09%) corresponded to 445,793 repetitive elements. N50, L50 and complete BUSCOs reached 1.02 Mb, 91 contigs and 97.1%, respectively. We predicted that the genome of *T. angustula* comprises 17,459 protein-coding genes and 4,108 non-coding RNAs. The mitogenome consisted of 17,410 bp, and all 37 genes were found to be on the positive strand, an unusual feature among bees. A phylogenomic analysis of 26 hymenopteran species revealed that six odorant receptor orthogroups of *T. angustula* were found to be experiencing rapid evolution, four of them undergoing significant contractions.

**Conclusions:**

Here, we provided the first nuclear and mitochondrial genome assemblies for the ecologically and economically important *T. angustula*, the fourth stingless bee species to be sequenced with LR technology thus far. We demonstrated that even relatively small amounts of LR data in combination with sufficient SR data can yield high-quality genome assemblies for bees.

**Supplementary Information:**

The online version contains supplementary material available at 10.1186/s12864-024-10502-z.

## Background

In the midst of the current biodiversity crisis, it has become critical to improve our knowledge about bees, the main pollinating agents of the angiosperms [1, 2]. Bees play a paramount role in plant reproduction in natural and cultivated ecosystems and thus are pivotal for environmental conservation, sustainable development and global food security [3–5]. The group of highly-eusocial stingless bees (Apidae: Meliponini), in particular, are of great relevance for comprising more than 600 described species [6]. Stingless bees are distributed across tropical and southern subtropical regions of the globe and are remarkably diverse in tropical rainforests, where 15–1,500 active colonies can be found within a single square kilometer, each housing up to tens of thousands of workers that routinely forage for floral resources [7–9]. It is therefore safe to assume that they are prime pollination providers for the highest tree diversity found on the planet [10, 11]. Moreover, many species are also well adapted to a varied range of environments, including dry forests and savannahs [12, 13].

The practice of rational rearing of stingless bees (i.e., meliponiculture) for honey harvesting has been performed since their independent domestication in various parts of the world during precolonial times [14]. Furthermore, their use for pollination has been shown to increase crop yields when adequately managed [15], leading many species to play a crucial role in crop production, including coffee, mango, and açai palm, among many others [16].

Recent advances in nucleic acid sequencing technologies and big data analyses have made it possible to sequence the genomes of bees, using only fractions of the human and financial resources employed in the sequencing of *Apis mellifera* Linnaeus nearly 20 years ago [17]. Today, genomic data can be used to investigate the genetic basis of key aspects in bee biology, including social behavior, pollen diet, and brood parasitism [18–20]. Genomic data have also been used to understand how past environmental and population dynamics may have shaped the contemporary genetic diversity of broadly distributed bees, leading to practical conservation guidelines [21–23]. The majority of these important studies, however, have focused on either honeybees or bumblebees, while comparatively little attention has been given to stingless bees [but see 18, 24].

The use of genomic data has revolutionized our understanding of the natural world. In particular, understanding genomic phenomena such as the expansion and contraction of gene families, which comprise a set of genes that tend to exhibit functional similarity [25, 26], can provide relevant insights into functional aspects of organisms [27, 28]. For example, it has been demonstrated that the genomes of the German cockroach and house fly, among all sequenced insects, have the largest repertoires of sensory receptors within the insect pickpocket gene family, which are responsible for detecting certain environmental stimuli such as water and salt [29]. Today we also know that gene family changes have had major implications in the evolution of herbivory within Drosophilidae [30]. More recently, an interesting research on gene families in megachilid bees showed that the species of the tribes Osmiini and Dioxyni have certains CYP9Q-related P450 enzymes that are able to detoxify the neonicotinoid insecticide thiacoprid, unlike their close relatives of the tribe Megachilini [31].

*Tetragonisca angustula* (Latreille), commonly known in Brazil by the Tupi-Guarani name “jataí” (= little bee), is the most commonly reared stingless bee species in Brazil. This is relevant because the country houses over 40% (~ 250 spp.) of all known stingless bees worldwide [32]. *Tetragonisca angustula* ranges from Rio Grande do Sul in southern Brazil to as far north as Chiapas in México [33], occupying a myriad of environments—from large forest fragments to major urban centers [34, 35]. The species can build natural nests in virtually any small cavity and collect resources from a broad range of plant groups [9, 36], which likely explains (at least partially) its remarkable adaptive success. *Tetragonisca angustula* has been a favorite among Brazilian stingless beekeepers because its management is relatively straightforward, workers are docile and its honey has a comparatively high market value [37, 38]. Today, many households across Brazil rely on the commercialization of honey and colonies of *T. angustula* as an important source of income [39, 40].

Despite its ecological and economic relevance, *T. angustula* has never been studied at the genomic level. Herein, we provide the first genome assembly for this small yet remarkable neotropical stingless bee. This genome sequence might provide scientific, economic, and ecological benefits. For instance, it might contribute to the advancement of the fields of bee sociogenomics and evolution [41], to the elaboration of population genetics-based conservation strategies [42], and to development of genomic tools that could potentially be used to identify genotypes susceptible to stressors (e.g., pesticides), allowing selection of colonies with improved health [43].

## Materials and methods

### DNA short-read sequencing and processing

We captured 34 males of *T. angustula* from an aggregation (close to a recently colonized trap nest) using a hand net within the municipality of Porto Seguro, Bahia state, Brazil (-16.4208, -39.0999). The individuals were transferred to a Styrofoam box covered with a fine mesh, transported alive to the laboratory, and placed in a -20 °C freezer for approximately 5 min. After this period, we removed their metasoma (to avoid possible contamination by gut microbiota) using flame-sterilized forceps, and finally stored the remaining body parts in a -80 °C freezer until further processing.

High molecular weight DNA was extracted using a Promega Wizard Genomic DNA Purification Kit. First, the males were divided into two 1.5 mL microtubes and ground with prechilled polypropylene pestles. We proceeded by following the manufacturer’s protocol, except that DNA was diluted in 50 µL of DNA Rehydration Solution for higher concentration. The library was prepared with an Illumina DNA Prep Kit using 1 µg of purified DNA. The library preparation, containing inserts of ~ 350 bp in size, was then sequenced through two different reactions, both on Illumina NextSeq 2000 platforms at Centro de Genômica Funcional (ESALQ/USP). The first was performed using an Illumina NextSeq 2000 P2 Reagents v3 Kit over 200 cycles, generating paired-end reads of ~ 101 bp in length (henceforth SR1). The second was performed with an Illumina NextSeq 2000 P2 300 M Reagents Kit over 600 cycles and produced paired-end reads of ~ 301 bp in length (henceforth SR3). The quality of both sequencings (SR1 and SR3) was assessed with FastQC v.0.11.9 [44].

Using the newly sequenced reads of SR3, we generated a separate single-end read dataset (henceforth SR2) with PEAR v.0.9.11 [45]. This software overlaps paired-end reads from a target fragment and merge them to increase the overall read length. Further, it allows for correction of the last bases, which are typically of lower quality than the first bases (especially in 301-pb reads). The input reads with an overlap < 10 bp and/or phred quality score < 20 were filtered out during the process of generating SR2.

### DNA long-read sequencing

Two females of *T. angustula* were used for PacBio HiFi long-read (LR) sequencing. They were sampled from the same natural nest located at the Instituto de Biociências, Universidade de São Paulo, Brazil (-23.5661, -46.7303). Both were captured while leaving the nest with a collection tube, which was then kept at -20ºC until subsequent procedures. High molecular weight DNA extraction was performed with a Promega Wizard® HMW DNA Extraction Kit. The entire bodies of the sampled females were macerated to ensure maximum yield. The DNA extraction followed the manufacturer’s protocol, but with two adaptations: we used both ethanol and isopropanol at -20ºC and stored the tubes also at -20ºC for the DNA precipitation. These adaptations improved the extraction yield, resulting in a higher DNA concentration at the end. The DNA extract was analyzed with a NanoDrop and then shipped to Macrogen (South Korea) for library preparation and sequencing.

The library was constructed using the protocol of SMRTbell™ Template Preparation, containing inserts from ~ 250 to 20,000 + bp. The sequencing was performed on a Pacific Biosciences (PacBio) Sequel II platform and its quality was assessed with LongQC v.1.2 [46].

### Transcriptome assembly

We obtained nine publicly available RNA sequencing (RNAseq) datasets of *T. angustula* from NCBI/GenBank (Supplementary Table S1) to be used as expressed sequence tag evidence in the later gene prediction analysis. They consisted of three triplets of RNAseq, each generated from individuals in one of the following developmental stages: larva, nurse, and forager.

All RNAseq data were used in combination to assemble a single wide-spectrum transcriptome with Trinity v.2.15.1 [47]. The function ‘-trimmomatic’ was activated to remove multiplexing tags and adaptor sequences aiming at improving assembly performance [48]. We assessed the quality of the assembled transcriptome employing two statistics: Contig Nx and Contig ExN50. The former was calculated with the perl script *TrinityStats.pl*. For the latter, we first estimated transcript abundance with *align_and_estimate_abundance.pl* using the alignment-free method Salmon [49], then generated a matrix of expression values with *abundance_estimates_to_matrix.pl* and finally calculated Contig ExN50 with *contig_ExN50_statistic.pl*. We also performed BUSCO v. 5.4.6 [50] to assess transcriptome completeness using the hymenoptera_odb10 database, which comprises 5,991 single-copy orthologs. Finally, we filtered the transcriptome to retain only the transcripts with at least a minimal expression across samples (‘--min_expr_any 1’), based on the matrix previously generated.

### Cross-species contamination and genome size estimation

All DNA reads were aligned against the human (NCBI accession GCF_000001405.40) and prokaryote genomes (NCBI RefSeq v.220) with Magic-BLAST v.1.7 [51] to eliminate possible sequencing contaminants. Filter-passed SAM reads were BAM-sorted and then FASTQ-converted with SAMtools v1.13 [52] for subsequent procedures. Filter-retained reads were manually blasted against the NCBI database for identification. The list of contaminant species/groups and their sequences were analyzed with the R [53] tools DECIPHER [54] and rentrez [55] to translate sequences and perform blast searches against the NCBI databases, respectively.

After the elimination of contaminants, we calculated the k-mer frequency distribution with Jellyfish v.2.3 [56] using SR1. Then, we estimated the size, heterozygosity rate and repetitive content of the genome of *T. angustula* by analyzing the produced histogram with the GenomeScope v.2.0 online tool [57], based on k-mer size and maximum coverage of 21 and 1,000, respectively.

### Genome and mitogenome assemblies

We followed a comprehensive data exploration approach aiming at finding an optimal *de novo* genome assembly for *T. angustula.* In all strategies, we used SR1 plus either SR2 or SR3 in combination with the LRs (Table 1). First, we executed MEGAHIT v.1.2.9 [58] with default parameters. We then conducted a series of analyses with MaSuRCA v.4.1.0 [59] testing its various built-in assembly algorithms, namely, Celera Assembler [60], Flye [61] and SOAPdenovo [62]. We also attempted to perform the widely used program SPAdes v3.15.5 [63], but analyses failed repeatedly due to computational resource limitations. Basic metrics (e.g., N50 and L50) and genome completeness of all preliminary assemblies were assessed with the perl script *scaffolds_stats.pl* [64] and BUSCO, respectively. Next, the best assemblies were polished with NextPolish v1.4.1 [65], and subsequently scaffolded by first using the LRs with the MaSuRCA built-in tool SAMBA [66] and then using SR1 with SSPACE v.3.0 [67]. Both programs were combined to fill the existing gaps and increase the contiguity of the assemblies. Finally, we performed *scaffolds_stats.pl* and BUSCO with the final assemblies to check the efficiency of the various strategies.

**Table 1:** Quality metrics of the various genome assemblies. The asterisk (*) indicates the preferred assembly. BUSCO refers to the percentage of complete orthologs found

Strategy (preliminary)	Reads^1^	Assembly algorithm	Contig count	Genome length (Mb)	Longest contig (Mb)	N50 (kb)	L50	GC (%)	BUSCO^2^
Megahit1	SR2	MEGAHIT	14,520	282.71	0.44	57.59	1,403	37.2%	94.8%
Megahit2	SR3	MEGAHIT	18,028	284.16	0.32	38.39	2,043	37.8%	93.9%
Masurca1^2^	SR2	Celera Assembler	945	280.17	3.98	648.39	128	37.6%	96.8%
Masurca2	SR2	SOAPdenovo	28,748	279.79	0.17	16.99	4,542	37.6%	90.5%
Masurca3	SR3	Flye	2,220	279.62	1.07	194.57	447	37.6%	95.3%
Masurca4^2^	SR3	Celera Assembler	967	284.17	2.50	720.47	118	37.6%	97.1%

Strategy (final)	Reads	Scaffolding algorithm	Contig count	Genome length (Mb)	Longest contig (Mb)	N50 (kb)	L50	GC (%)	BUSCO^2^
Masurca1			945	280.17	3.98	648.39	128	37.6%	96.8%
Samba Sspace	LRs SR1	SAMBA SSPACE	790 675	280.58 280.60	3.98 4.97	817.18 902.84	106 100	37.6% 37.6%	96.8% 96.8%
Masurca4			967	284.17	2.50	720.47	118	37.6%	97.1%
Samba Sspace^*^	LRs SR1	SAMBA SSPACE	829 683	284.45 284.49	2.75 3.47	951.11 1,021.56	100 91	37.6% 37.6%	97.1% 97.1%

^1^SR1 and LRs were also used in all preliminary strategies (see text)

^2^Best preliminary strategies

The mitogenome of *T. angustula* was assembled through a two-step process. First, a preliminary *de novo* assembly was performed with GetOrganelle v.1.7.7.0 toolkit [68] using SR2. We then used the *de novo* assembly as reference to produce a final assembly with the MitoHifi v.3.0.0 pipeline [69] using the LRs. The final assembly was annotated on the MITOS2 web server [70], using the invertebrate genetic code (code 5) and RefSeq 89 Metazoa as reference. Manual verification and refinement of gene positions were carried out with BLAST+. The final assembly and coverage plots from both analyses were visualized on the Proksee web server [71].

### Repetitive element masking and gene prediction

First, we employed the program RepeatModeler v.2.0.2 [72] to generate a species-specific repetitive library for *T. angustula* based on both the Dfam v.3.6 [73] and RepBase v.20,181,026 [74] databases. The identified elements were then masked in the assembled genome with RepeatMasker v.4.1.2 [75].

Protein-coding genes in the repeat-masked genome were predicted with the program BRAKER3 [76] using both the GeneMark-ETP [77] and Augustus [78] predictors. First, the RNAseq data were aligned against the genome assembly with StringTie v2.2.1 [79] and then the bam files were used as evidence in the predictions.

### Gene functional annotation

The predicted protein-coding genes were functionally annotated based on sequence homology using Diamond v.2.0.14 [80], eggNOG-mapper v.2.1.12 [81] and InterProScan v.5.63-95 [82]. First, we aligned the protein-coding sequence data against the UniProtKB/Swiss-Prot [83] with Diamond/BlastX. Next, we produced a separate FASTA with the sequences that did not match Swiss-Prot with SeqKit v.2.2 [84] and aligned them against the UniProtKB/TrEMBL database [83] through another round of Diamond/BlastX. Both Diamond outputs were manually filtered (‘sort -k1,1 -k12,12nr -k11,11n | sort -k1,1 -u’) to retain only the best hits for downstream annotation analyses. Finally, we performed InterProScan with the protein sequences as queries to identify protein domains (15 databases), gene ontology (GO database), and biological pathways (MetaCyc and Reactome databases).

In addition to protein-coding genes, we also identified non-coding RNA elements (ncRNAs) with Infernal v.1.1.5 [85] based on the Rfam v.14.9 database [86]; transfer RNAs (tRNAs), in particular, were identified with tRNAscan-SE v. 2.0.9 [87].

### Orthogroup evolution and phylogenomic analyses

We followed a homology-based comparative genomic approach to infer changes (gains and losses) in orthogroups within an evolutionary framework. For this analysis, we included *T. angustula* plus 25 other hymenopteran species, whose protein sequence data were obtained from NCBI/GenBank (Supplementary Table S2). First, we performed OrthoFinder v.2.5.5 [88] with default parameters for ortholog identification and clustering. We carried out BUSCO to extract single-copy orthologs, which were subsequently aligned separately with MUSCLE v.3.8.1551 [89], end-trimmed with trimAL v.1.4.1 [90], and then amalgamated into a 90% completeness matrix, using the pipeline BUSCO Phylogenomics [91]. Next, we inferred a maximum likelihood (ML) tree with IQ-TREE v. 2.1.3 [92] through 1,000 ultrafast bootstrap replicates [93]. We invoked the ‘--symtest-remove-bad’ option to exclude the single-copy orthologs violating stationarity, reversibility and homogeneity assumptions to avoid inference biases. ModelFinder was used to select the best partitioning scheme and substitution models based on a greedy strategy [94]; however, only the top 33% merging schemes were assessed to save computational time. The resulting phylogram was subsequently converted to a time-calibrated ultrametric tree with r8s v.1.81 [95], using four calibration points obtained from TimeTree 5 [96]: root, 240 million years ago (Mya); Apocrita, 179 Mya; bees, 110 Mya; and corbiculates, 82 Mya. Finally, we used FigTree v.1.4.4 [97] for visual inspection and final annotation of trees.

We inferred the number of orthogroup changes (contractions and expansions) for each node and tip of our ML tree using CAFE v. 4.2.1 [98]. This was achieved by estimating a mean birth-death parameter (lambda), which represents the likelihood that any ortholog will be gained or lost based on orthogroup counts and dated cladogenetic events. We estimated lambda with *cafe.py*, summarized the computes with *cafetutorial_report_analysis.py*, and finally plotted them onto the phylogeny with *cafetutorial_draw_tree.py*.

## Results

### Raw data from the LR and SR sequencings and respective coverages

With the PacBio DNA sequencing, we generated 366,533 LRs comprising 2.31 Gb (8-fold coverage); mean size and N50 were 10.66 kb and 11.74 kb, respectively (Supplementary Fig. S1). With the Illumina DNA sequencings, we generated 345,692,872 SRs consisting of 45.70 Gb (161-fold coverage), including 276,666,288 reads (27.62 Gb) from SR1 and 69,026,584 reads (18.07 Gb) from SR3 (Supplementary Figs S2–S5). To generate SR2, 1,061,968 paired-end reads (0.25 Gb) were discarded from SR3, and the filter-passed reads were merged into 33,982,308 single-end reads (9.70 Gb).

### Transcriptomic evidence for protein-coding gene annotation

The nine RNAseq datasets for *T. angustula* analyzed by us included 577,856,230 reads, comprising 47.32 Gb (Supplementary Table S1). Read count and dataset size ranged from 27,737,128 to 41,105,492 reads (mean of 32,103,123 ± 5,237,347 reads) and from 1.62 to 3.27 Gb (mean of 2.62 ± 0.45 Gb), respectively, across datasets.

After filtering out the transcripts that did not achieve the expression threshold (--min_expr_any 1, *n* = 55,910), the *de novo* transcriptome of *T. angustula* comprised 138,341 assembled transcripts (GC 37.4%), totaling 221.88 Mb. The longest transcript, N50, and L50 were, respectively, 38.04 kb, 4.02 kb and 15,657 (Supplementary Fig. S6 and Supplementary Table S3). Our BUSCO assessment of the transcriptome returned 5,906 complete single-copy orthologs, thus yielding a completeness ratio of 97.2%.

### A 284-Mb genome with nearly 17,500 protein-coding genes

Based on the k-mer frequency distribution of reads, we estimated the genome of *T. angustula* to be 317.60–317.92 Mb in size, of which 256.01–256.26 Mb (80.6%) and 61.59–61.65 Mb (19.4%) corresponded to the unique and repetitive regions, respectively (Supplementary Table S4). The estimated overall heterozygosity rate was 0.27%, with a distinct peak occurring at the k-mer coverage of 13.3 (Supplementary Fig. S7).

In total, ten genome assemblies were produced, and all of which were considerably smaller than previously estimated (Table 1). They were nonetheless fairly similar in size, ranging from 279.62 to 284.49 Mb (mean of 282.09 ± 1.99 Mb). Similarly, the calculated GC content was consistently around 37% irrespective of the strategy used. However, the number of contigs greater than 1 kb and the length of the longest contig varied substantially across strategies, ranging from 675 to 28,748 (mean of 572.04 ± 404.58) and from 0.17 to 4.97 Mb (mean of 2.51 ± 1.58 Mb), respectively. The N50 varied from 16.99 to 1,021.56 kb (mean 519.67 ± 348.86 kb), the L50 varied from 91 to 4,542 contigs (mean of 111.99 ± 111.59 contigs) and BUSCO completeness reached between 90.5 and 97.1% (mean of 95.84 ± 1.90%). Based on the assessed parameters, MaSuRCA yielded better preliminary assemblies than MEGAHIT, except when SOAPdenovo was employed within the former. The two best strategies used either SR2 (Masurca1) or SR3 (Masurca4), and both were carried out with Celera Assembler. Our preferred assembly strategy (i.e., Masurca4 + NextPolish + Samba + SSPACE) resulted in 683 contigs comprising 284.49 Mb (GC 37.6%), with N50 of 1,021 Mb, L50 of 91 contigs and BUSCO completeness reaching 97.1%.

Prior to gene prediction, we identified and masked 445,793 repetitive elements, which together comprised 62.84 Mb and corresponded to 22.09% of the genome of *T. angustula* (Supplementary Table S5). Most repetitive elements were interspersed (*n* = 215,530 or 52.11 Mb, 48.34%), followed by simple (*n* = 196,861 or 8.97 Mb, 44.16%) and low-complexity repeats (*n* = 33,402 or 1.74 Mb, 7.5%).

We predicted that the genome of *T. angustula* comprises 17,458 protein-coding genes totaling 33.94 Mb, which corresponded to 11.9% of the complete genome. We annotated the biological function of 16,032 protein-coding genes (91.8% of the total), including 10,913 annotations (68.1%) based on Swiss-Prot and the other 5,119 (31.9%) based on TrEMBL (Supplementary Table S6). Using InterProScan, we also functionally annotated 16,581 protein-coding genes (94.9% of the total) by associating them with 94,059 protein domains, 22,975 biological pathways and 10,973 GO terms previously identified. Also, 15,693 protein-coding gene sequences matched the eggNOG database. Finally, we identified 4,108 ncRNA genes (Supplementary Tables S7 and S8), most of which were micro RNAs (*n* = 1,535, 37.3%), followed by small nuclear RNAs (*n* = 812, 19.8%), small RNAs (*n* = 764, 18.6%), cis-regulatory RNA elements (*n* = 519, 12.6%), and transport RNAs (*n* = 178, 9.4%).

### The mitochondrial genome with the longest A + T region among stingless bees

**Fig. 1:**
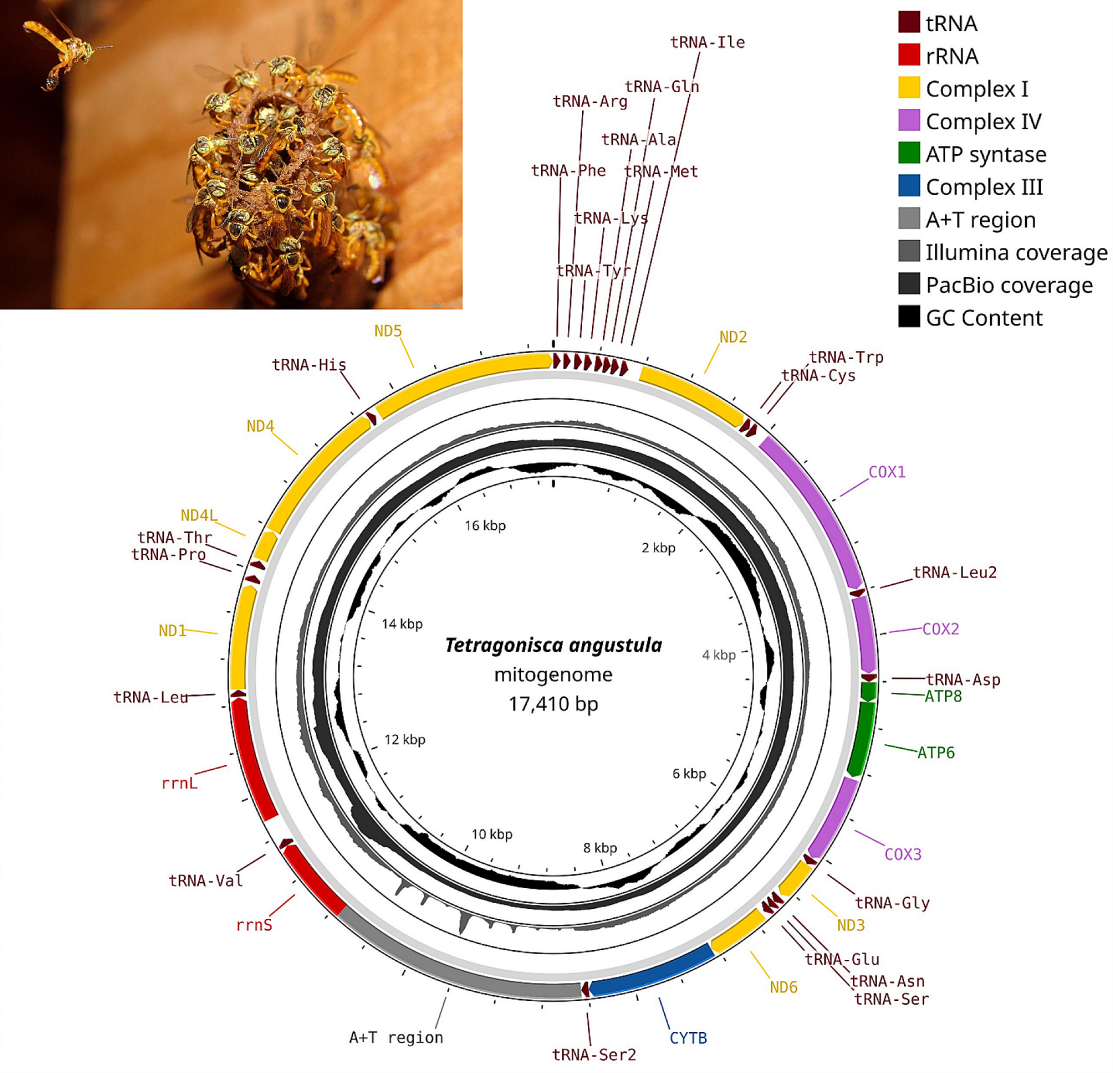
Circular schematic representation of the complete mitogenome of *Tetragonisca angustula*. From the outer to inner circle: the arrows indicate gene directions, coverage plot of the Illumina SR alignment, coverage plot of PacBio HiFi LR alignment and GC skew plot. Photo of *T. angustula* by Cristiano Menezes

**Fig. 2:**
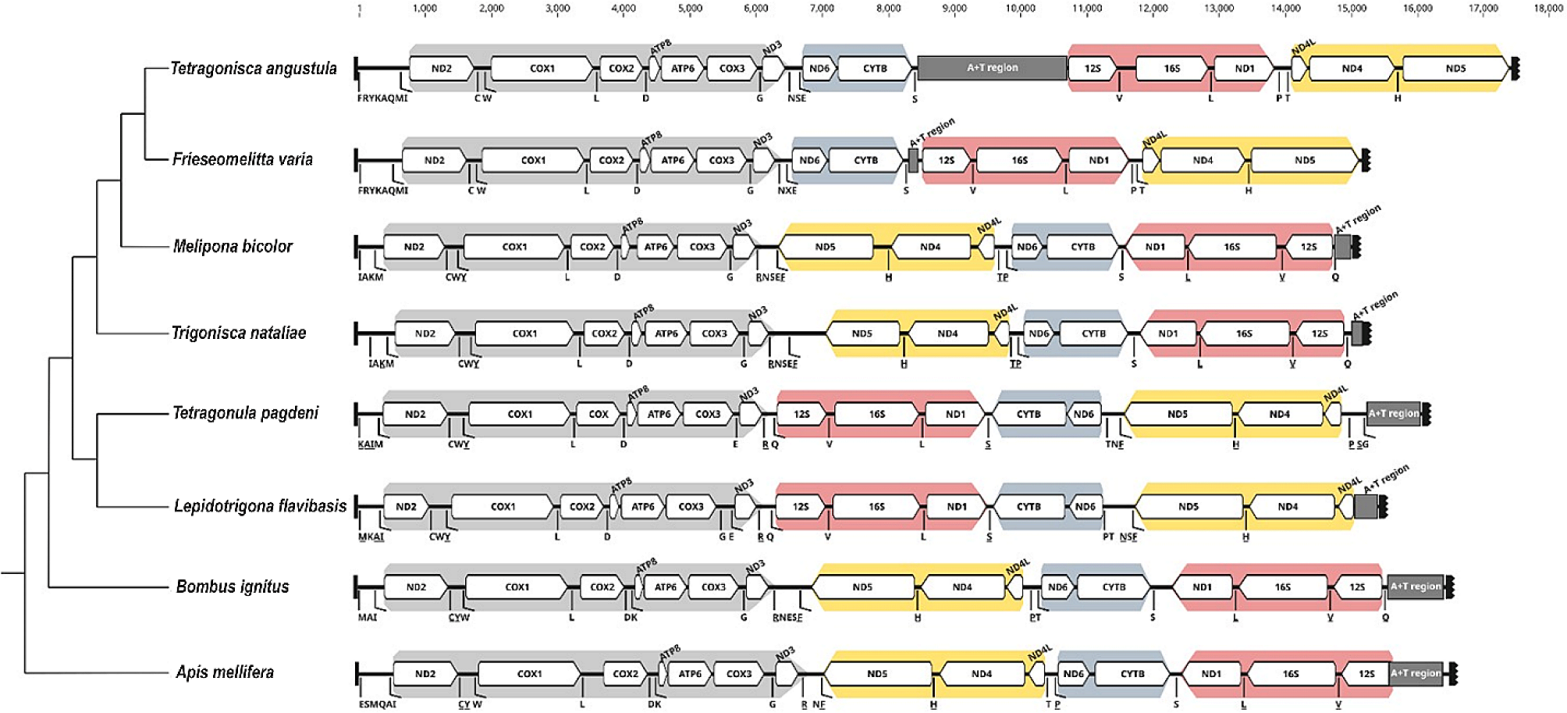
Linear schematic representation of the mitogenomic organization in *Tetragonisca angustula* compared to other corbiculate bees. Colors indicate conserved blocks of protein-coding genes that maintain the same organization even after rearrangement events

The mitogenome of *T. angustula*, a circular double-stranded DNA molecule spanning 17,410 bp, encodes a set of 37 genes, including 2 rRNAs, 22 tRNAs and 13 protein-coding genes (Fig. 1). All genes were identified on the putative plus strand, as in *F. varia*. Moreover, the organizational pattern of both mitogenomes are identical and seemingly unique among corbiculate bees (Fig. 2).

The mitogenome of *T. angustula* includes the longest A + T-rich region among all stingless bee species sequenced to date, encompassing 2,276 bp. Putative replication origin stems, accompanied by flanking TA dinucleotide repeats, were identified. A significant decrease in coverage from LRs and, especially, from SRs, was observed for this region, with the latter also showing low coverage uniformity. Conserved motifs like T(n) (polyT stretch) and putative stop signals, previously reported for other bee species [99, 100], were not found.

### Contaminants are mostly bee gut bacteria

Our search for contaminants found ten bacteria species often associated with bees (Supplementary Table S9). The most abundant was *Gilliamela apis*, a common bacteria of bee guts. A gene of the bacteria *Pantoea agglomerans* was also identified; this species is usually associated with *A. mellifera*, typically in honey sacs. The set of species included gram-positive and gram-negative bacteria that are usually implied in bee intestinal functions.

### Late-miocene divergence between *Tetragonisca angustula* and *Frieseomelitta varia*

Our final data matrix included 310 single-copy orthologs comprising a total of 109,967 sites (354.7 sites/ortholog on average). These consisted of 39,990 parsimony-informative sites (36.4%), 19,905 singletons (18.1%) and 50,072 non-variable sites (45.5%%).

**Fig. 3:**
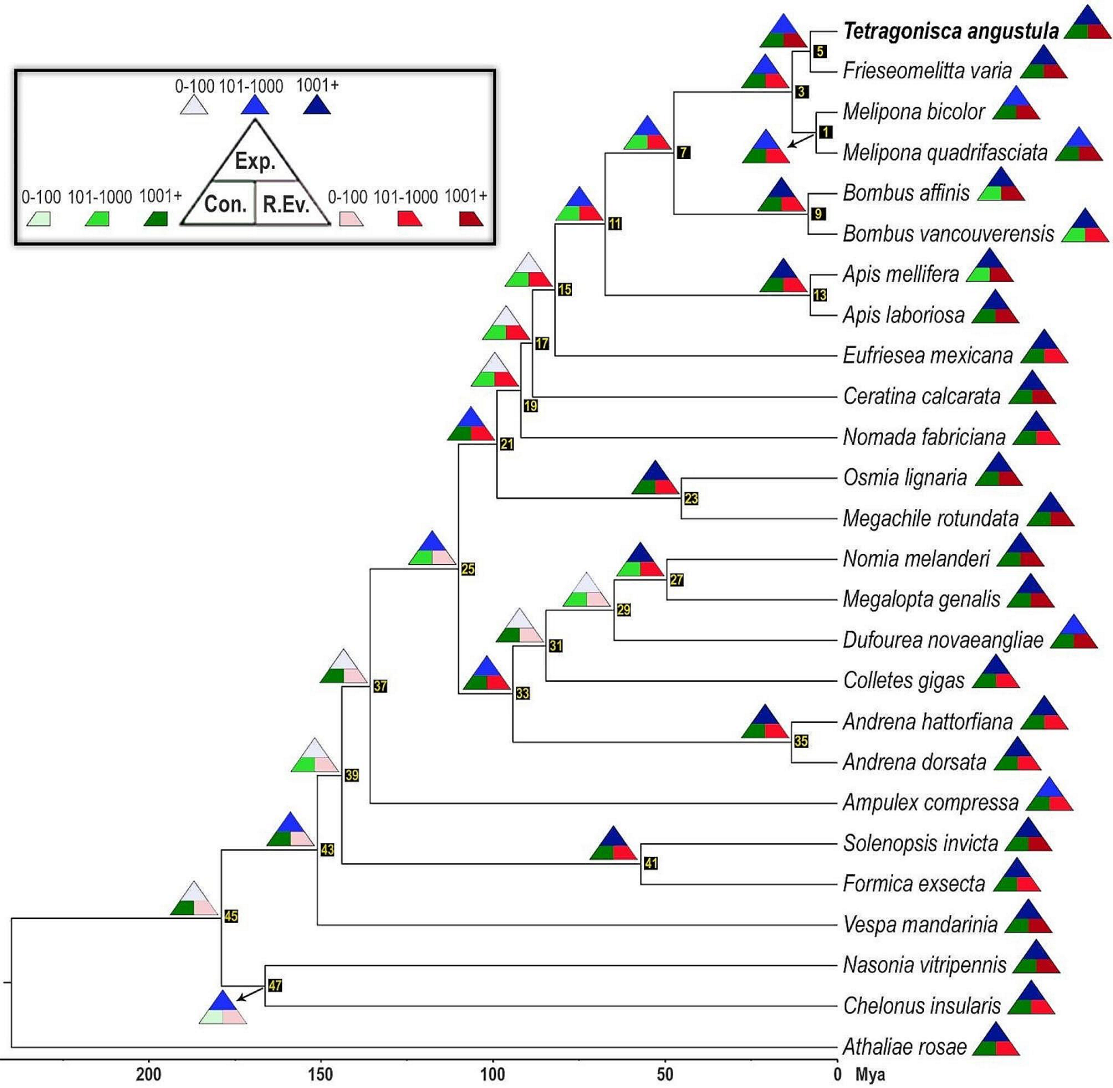
Dated ML tree inferred from USCO data showing the phylogenetic placement of *Tetragonisca angustula* (bold) within Hymenoptera. Color-coded triangles depict the approximate number of orthogroups in expansion (shades of blue), contraction (shades of green) and rapid evolution (shades of red) for the corresponding internode or terminal species. The numbers inside the small black rectangles indicate the clade numbers attributed by CAFE. All clades were recovered with maximum support (omitted)

The ML analysis yielded a fully resolved phylogenomic tree (Fig. 3) in which all nodes were maximally supported. The inferred high-level relationships were fully consistent with those from previous phylogenomic studies, including the following: (1) short- and long-tongued bees were recovered as reciprocally monophyletic in the absence of Melittidae; (2) Colletidae and Halictidae appeared as sister taxa, and together they comprised the sister group to Andrenidae; and (3) corbiculate bees formed a monophyletic clade within Apidae. Stingless bees and bumblebees, which were recovered as reciprocally monophyletic, likely diverged from each other 49.3 Mya in the mid-Eocene, according to our dating analysis. *Tetragonisca angustula* would have diverged from its closest relative, *F. varia*, 7.1 Mya during the late-Miocene, a relatively late divergence when compared to that of the congeneric species *B. affinis* and *B. vancouverensis* Cresson (8.8 Mya).

### Odorant receptor orthogroups show significant contractions

OrthoFinder identified 590,881 orthologs and assigned 569,733 (96.4%) of them to 21,462 orthogroups, yielding an average of 26.54 orthologs per orthogroup (Supplementary Table S10). Of these, 5,612 orthogroups (26.1%) were shared by all species, while 3,530 (16.4%) were species specific; the latter comprised 2.7% (*n* = 16,199) of the assigned orthologs. *Tetragonisca angustula*, in particular, had 16,712 of its 17,519 orthologs (95.4%) assigned to 10,193 orthogroups (1.64 ortholog/orthogroup), including 26 orthogroups and 62 orthologs that were found to be specific to the species.

We inferred 247,680 events in which an orthogroup underwent a change in size, including 178,891 contractions and 68,789 expansions (Supplementary Table S11), based on the lambda value (0.00882247) calculated by CAFE. However, 5,364 of the identified orthogroups (25%) did not change significantly in terms of gene composition (*p* > 0.05). *Tetragonisca angustula* accounted for 2,878 of the 247,680 change events, 1,712 of which (59.5%) represented contraction events. Overall, the number of contractions was significantly higher than that of expansions (*F* = 13.6004, *p* = 0.0003712) among the sampled hymenopterans. Neither the mean number of contractions (*F =* 0.8342, *p* = 0.5553) nor of expansions (*F =* 3.8036, *p* = 0.06292) was significantly different between bees and non-bees. The same was found when stingless bees and the other bees were compared with each other: contractions (*F =* 0.8247, *p* = 0.3765) and expansions (*F =* 0.6478, *p* = 0.4320).

CAFE also identified 41,802 events of rapid evolution spanning 14,010 different orthogroups, 1,485 of which were found in *T. angustula*, including 843 (56.8%) in expansion and 642 (43.2%) in contraction. At least six of these rapidly-evolving orthogroups comprised odorant receptors (ORs)—which are found in the membranes of olfactory neurons in insects—including two orthogroups in expansion and four in contraction (Supplementary Table S12). Overall, seven OR orthologs were gained and 22 were lost by *T. angustula*, leaving a negative balance of 15 OR orthologs. In contrast, ten rapidly evolving OR orthogroups were predicted for *F. varia*, eight of them in expansion (+ 37) and only two in contraction (-4), resulting in a balance of 33 more OR orthologs. Both *M. bicolor* (+ 7) and *M. quadrifasciata* (+ 1) as well as the inferred ancestors of Meliponini (+ 2) and *T. angustula* plus *F. varia* (+ 8) gained more OR orthologs than lost.

## Discussion

Stingless bees play a vital role for humankind. They are raised for honey production and provide crucial ecosystem services by pollinating the natural flora and commercial crops [101]. They also promote environmental friendly strategies and occupational therapy [102, 103]. Therefore, gaining knowledge on stingless bees and, in particular, understanding the importance of genomic structures and processes in their biology and behavior, as has been widely performed with *A. mellifera* [104–106], has become increasingly important. Unfortunately, very few stingless bees have so far had their genomes sequenced [107]. As applied genomic research is totally reliant on genomic data, this current shortage becomes a pressing issue that demands attention. Therefore, providing the first genome assembly for *T. angustula*, a key species of the Brazilian bee fauna, was the main contribution of the present work to the incipient field of stingless bee genomics.

Besides its unparalleled ecological and economic relevance, *T. angustula* also stands out among neotropical stingless bees for having a unique combination of biological features. First and foremost, *T. angustula* is extremely adaptable to varied habitats, including fully urbanized areas, likely due to its broad pollen diet and nesting substrate use, high reproduction rates and great ability to maintain vital procedures within the colony (e.g., brood cell construction and egg laying) even in unfavorable conditions [15, 34]. Second, *T. angustula* is one of the few stingless bee species whose queens are known to mate with multiple males during nuptial flight [108; but see 109]. In fact, multiple mating by females has been shown to be a relatively rare phenomenon among social hymenopterans, despite the fact that it is known to increase genetic diversity of the offspring [110]. Third, *T. angustula* is less aggressive than other stingless bees (e.g., *Scaptotrigona*) and its workers usually retreat back to the nest when disturbed by humans and other vertebrates, although they effectively defend the colony against robber bees of the genus *Lestrimelitta* [34]. In fact, the defense strategy of *T. angustula* against robber bees is perhaps one of the most distinguishing biological features of this bee as it likely led to the origin of a new caste of workers with specialized morphology to guard the nest [111].The genome assembly provided herein is an important step towards the understanding of the genomic mechanisms behind these and other aspects of the intriguing biology of stingless bees.*Tetragonisca angustula* is the fourth stingless bee species to have its nuclear genome sequenced using an LR-based sequencing platform. Although SR-based technologies have transformed how we can investigate the natural world from a genomic perspective, they have nonetheless shown severe limitations in detecting, for example, repetitive regions and gene duplications [112]. The use of LRs in the present study exemplifies how this technology can result in higher assembly quality metrics, when compared to previous stingless bee genomes assembled based only on SRs (Supplementary Table S14). On the other hand, the same metrics were not as performant as the ones from the LR-assembled genomes of *M. bicolor* and other eusocial bees [113–115]. One possible explanation for these results is that we could only generate 2.31 Gb worth of LRs, which resulted in a low sequencing coverage (8-fold). This nonetheless did not prevent us from achieving a high-quality assembly when LRs and SRs were combined and thoroughly analyzed, thus demonstrating that even low-coverage LR data can yield satisfactory results.

The use of LRs also enabled us to assemble the mitogenome of *T. angustula* with greater completeness compared to when only SRs were employed. This can be primarily ascribed to the A + T-rich region, which is typically challenging to identify due to its repetitive and low complexity features [116]. However, a decrease in coverage was also observed for the LRs, potentially indicating the occurrence of intraindividual variation in the A + T-rich region (i.e., length heteroplasmy). This phenomenon has previously been reported for multiple species across the Animal Kingdom [117–121], including bees [122], suggesting that it may be a widespread trait. We also found that the mitogenome of *T. angustula* shares the same gene organization with that of *F. varia* [24], which distinguishes both from the other sequenced species of stingless bees. Considerable rearrangements have been observed in stingless bees [123, 124], albeit with some gene blocks seemingly maintaining their conserved organization—another phenomenon commonly observed in metazoans [125]. However, only *T. angustula* and *F. varia*, as far as the available data show, exhibit the peculiarity of having all mitochondrial genes encoded on the putative plus strand. With this regard, the emergence of LR sequencing methods may be a turning point for a better understanding of the evolutionary dynamics of stingless bee mitogenomes.

The genome of *T. angustula* (284.49 Mb) is larger than the observed average size (277.7 Mb) for stingless bees, when the highly fragmented genome of *Lepidotrigona ventralis* (127,582 contigs) is not considered. This may be due to an unusually large repetitive content—62.8 Mb or 22.1% of the genome—even in comparison with that of *F. varia* (39.1 Mb or 14.2% of the genome) [24]. The number of protein-coding genes found in the genome of *T. angustula* (17,458) is also higher than previously predicted for the stingless bee species sequenced so far, except *M. bicolor* (20,278 protein-coding genes), although this number may be overestimated [113]. Moreover, none of the other stingless bee genomes sequenced to date was annotated using transcriptomic evidence from multiple life stages as in the present study. It is nevertheless important to ponder that the procedures adopted for repeat masking and gene prediction herein and previously differed considerably [18, 24, 113], meaning that direct comparisons must be interpreted with caution.

The bacteria profile found in the sequencing of *T. angustula* closely aligns with the ones previously reported for other corbiculate bees, such as *(A) mellifera* and *(B) terrestris*. Among the species identified, *Gilliamella apis*, primarily located in the bee midgut, plays a crucial role in the digestion of pollen and pectin [126]. Additionally, *Snodgrassella comunis* and *S. alvi* are associated with the metabolism of various carbohydrates and also seem to have coevolved with honeybees and bumblebees [127]. These findings show that, despite the intrinsic and intricate biology of each bee species [128], similar bacterial profiles may emerge, particularly those linked to the digestion of macromolecules.

Our orthogroup analysis indicated that *T. angustula* has lost more orthologs than gained over the course of its evolutionary history. However, one must keep in mind that orthogroup identification and clustering is totally reliant on the premises that (1) the genome has been fully assembled, and that (2) all protein-coding genes have been correctly predicted, in upstream analyses. In other words, we simply cannot rule out the possibility that we may have failed in our attempt to identify a series of orthologs that are actually present in the genome of *T. angustula*. In fact, it is important to emphasize that 3% of the orthologs (*n* = 180) searched for in the BUSCO analysis could not be found.

An ortholog may be lost, for example, when deleterious mutations are accumulated, without subsequent negative selection [129], after the positive pressure to retain it has ceased [130] or simply if it can be functionally replaced by another ortholog [131]. Perhaps the most notable finding regarding ortholog loss in our study was that four of the orthogroups found to be experiencing rapid contraction included ORs, which are responsible for detecting volatile organic compounds in the environment [132]. In insects, ORs are predominantly found in sensory structures such as the proboscis, maxillary palpi and antennae [133], and mediates the search for food, oviposition sites, sexual mates, among other complex behaviors [134, 135]. These processes are so crucial for insect survival and reproduction that the origin of ORs has been shown to be a molecular synapomorphy of Insecta, likely emerging as an adaptation to terrestriality [136].

Therefore, the finding that OR orthogroups have been rapidly contracting in *T. angustula* is intriguing and raises important questions concerning its evolution. One could claim that eusociality may have played a role in the observed OR contraction, as previously shown regarding immunity [137] and regulatory orthologs [138]. However, our analyses actually revealed a rather opposite trend, i.e., a rapid expansion within Meliponini that was drastically reversed in *T. angustula*. It has been shown that the chemoreception in *T. angustula* is characterized by a notable size reduction of the neurological structures responsible for processing the information conveyed by these receptors [139]. Thus, we believe that the observed contraction may be ascribed to broad generalist lifestyle regarding flower hosts and nesting substrates. This hypotheses, however, can only be tested through a broader and more rigorous investigation, in terms of both data and taxa, which goes beyond the scope of the present paper.

## Conclusions

In this paper, we provided the first genome assembly for *T. angustula*, the most important stingless bee species in Brazil from an economic standpoint. Despite the enormous relevance of this important group of pollinators for tropical ecosystems, this is only the fourth stingless bee to be fully sequenced using a LR-based technology, as far as we are aware. Relatively low coverage LR data did not prevent us from obtaining a high-quality genome assembly, therefore validating its use in combination with high-coverage SRs.

Our phylogeny-based, comparative genomic analyses revealed an overall pattern of orthogroup contraction in *T. angustula*, notably in ORs. As these receptors are of paramount importance for insect survival and adaptation, our findings provide novel insights into the evolution of ORs within Meliponini and other groups of eusocial insects.

### Electronic supplementary material

Below is the link to the electronic supplementary material.

**Table S1**. Overview of the nine RNAseq datasets from three different life stages of *Tetragonisca angustula* obtained directly from NCBI

**Table S2**. Insect taxa included in each of our genomic analyses and the NCBI accessions for their data

**Table S3**. Contig Nx statistics, and other relevant metrics, of the *de novo* transcriptome of *Tetragonisca angustula* when all transcripts (2nd column) or only the longest isoforms (3rd column) are considered

**Table S4**. Estimations of the total size, and repetitive and unique lengths of the genome of *Tetragonisca angustula* based on a GenomeScope assessment

**Table S5**. Description of the repeat elements masked in the genome of *Tetragonisca angustula* for the purpose of gene prediction. 2nd column, total count; 3rd column, total number of bases masked; 4th column, relative size of the corresponding repeat element

**Table S6**. Databases against which our gene annotation analyses were conducted. The numbers under “Matches” correspond to those of predicted protein-coding gene sequences that aligned against the corresponding database

**Table S7**. Non-coding RNA genes (2nd column) classified by type (3rd column) that were identified in the genome of *Tetragonisca angustula*

**Table S8**. Transport RNA genes along with their bounds (3rd and 4th columns), type (5th column) and anti codon (6th column) that were identified in the genome of *Tetragonisca angustula*

**Table S9**. Contaminants (bacteria) found among the raw genome sequencing data of *Tetragonisca angustula*

**Table S10**. Orthogroup identification overview. 2nd column, number of orthologs identified by Orthofinder; 3rd column, number (and percentage) of identified orthologs that were assigned to orthogroups; 4th column, number (and percentage) of the identified orthologs that could not be assigned to any orthogroup; 5th column, number (and percentage) of orthogroups that were found in the genome of the corresponding species; 6th column, number of orthogroups that were found exclusively in the genome of the corresponding species; 7th column, number (and percentage) of the identified orthologs that were assigned to the orthogroups found exclusively in the genome of the corresponding species

**Table S11**. Overview of the predicted orthogroup changes,, expansions (2nd to 4th columns) and contractions (5th to 7th columns), for the genome of each insect species analyzed. Numbers between parenthesis in the 2nd and 5th columns indicate the number of orthogroups found to be rapidly expanding and contracting, respectively. Positive or negative numbers in the 9th column indicate an overall expansion or contraction, respectively

**Table S12**. Expansions (+) and contractions (-) in rapidly evolving odorant receptor orthogroups within Meliponini. “0” indicates that the orthogroup did not change in size for the corresponding species or node. <3>, ancestor of Meliponini; <5>, ancestor of *Tetragonisca angustula* plus *Frieseomelitta varia*; <1>, ancestor of *Melipona*

**Table S13**. Predicted number of orthogroups in expansion (2nd column) and contraction (3rd column) in for the genome of each sampled stingless bee species based on OrthoVenn analysis

**Table S14**. Genome profile of the stingless bee species sequenced to date and the NCBI accessions for their data

**Fig. S1**. Six quality parameters of the PacBio long-read sequencing obtained through a LongQC assessment

**Fig. S2**. Eight quality parameters of 2 × 101 (SR1) Illumina short-read sequencing (R1) based on FastQC analysis

**Fig. S3**. Eight quality parameters of 2 × 101 (SR1) Illumina short-read sequencing (R2) based on FastQC analysis

**Fig. S4**. Eight quality parameters of 2 × 301 (SR3) Illumina short-read sequencing (R1) based on FastQC analysis

**Fig. S5**. Eight quality parameters of 2 × 301 (SR3) Illumina short-read sequencing (R2) based on FastQC analysis

**Fig. S6**. Contig ExN50 plot of the *de novo*-assembled transcriptome of *Tetragonisca angustula* showing a peak at E95 of roughly 4.7 kb

**Fig. S7**. GenomeScope profile plots of absolute (top graph) and log-transformed (bottom graph) k-mer frequency distributions, at a k-mer length of 21, built based on the SR1 reads

## Data Availability

The raw reads from the PacBio HiFi sequencing are available at the NCBI Sequence Read Archive (SRA) database under the accession SRR26419207, within the BioProject PRJNA1029524 and BioSample SAMN37871898. The raw reads from the two Illumina whole-genome sequencings are available at the NCBI SRA database under the accessions SRR26374687 (SR1) and SRR26374686 (SR3), within the BioProject PRJNA970376 and BioSample SAMN35000760. The whole-genome and mitogenome assemblies are available at the NCBI Whole-Genome Sequencing database under the accessions JAWNGG020000000 and OR030859, respectively, within the BioProject PRJNA970376 and BioSample SAMN35000760.
