# Supplementary material for: The nuclear and mitochondrial genome assemblies of Tetragonisca angustula (Apidae: Meliponini), a tiny yet remarkable pollinator in the Neotropics
Source: BMC Genomics. 2024 Jun 11;25:587. doi: 10.1186/s12864-024-10502-z (PMC11167848; doi:10.1186/s12864-024-10502-z)
Supplement: Supplementary file 1 — Table S1. Overview of the nine RNAseq datasets from three different life stages of Tetragonisca angustula obtained directly from NCBI [file 12864_2024_10502_MOESM1_ESM.docx]

**Table S1** Overview of the nine RNAseq datasets from three different life stages of *Tetragonisca angustula* obtained directly from NCBI.

| Dataset | Replicate | Read count | Size (Gb) | BioProject | BioSample | Run |
| --- | --- | --- | --- | --- | --- | --- |
| Larva | 1 | 57,228,306 | 4.91 | PRJNA1029524 | SAMN37873776 | SRR26421937 |
| Larva | 2 | 76,393,886 | 6.54 | PRJNA1029524 | SAMN37873777 | SRR26422268 |
| Larva | 3 | 51,708,756 | 3.60 | PRJNA1029524 | SAMN37873778 | SRR26422305 |
| Nurse | 1 | 82,210,984 | 5.74 | PRJNA615177 | SAMN14447504 | SRR11440494 |
| Nurse | 2 | 55,474,256 | 4.74 | PRJNA615177 | SAMN14447504 | SRR11440495 |
| Nurse | 3 | 66,413,432 | 5.68 | PRJNA615177 | SAMN14447504 | SRR11440496 |
| Forager | 1 | 70,108,004 | 5.99 | PRJNA615177 | SAMN14447189 | SRR11426431 |
| Forager | 2 | 50,703,786 | 4.34 | PRJNA615177 | SAMN14447189 | SRR11426432 |
| Forager | 3 | 67,614,820 | 5.77 | PRJNA615177 | SAMN14447189 | SRR11426433 |
|  |  | **Total** = 577,856,230 | **Total** = 47.31 |  |  |  |
