# Supplementary material for: The nuclear and mitochondrial genome assemblies of Tetragonisca angustula (Apidae: Meliponini), a tiny yet remarkable pollinator in the Neotropics
Source: BMC Genomics. 2024 Jun 11;25:587. doi: 10.1186/s12864-024-10502-z (PMC11167848; doi:10.1186/s12864-024-10502-z)
Supplement: Supplementary file 2 — Table S2. Insect taxa included in each of our genomic analyses and the NCBI accessions for their data [file 12864_2024_10502_MOESM2_ESM.docx]

**Table S2** Insect taxa included in each of our genomic analyses and the NCBI accessions for their data.

| Species | Classification | Analysis | |
| --- | --- | --- | --- |
|  |  | Orthogroup evolution | Mitogenome synteny |
| *Ampulex compressa* | Apoidea: Apulicidae | GCA_019049445.1 | not applicable |
| *Andrena dorsata* | Apoidea: Apidae | GCA_929108735.1 | not applicable |
| *Andrena hattorfiana* | Apoidea: Apidae | GCA_944738655.1 | not applicable |
| *Apis laboriosa* | Apoidea: Apidae | GCA_014066325.1 | not applicable |
| *Apis mellifera* | Apoidea: Apidae | GCF_003254395.2 | NC_051932 |
| *Athalia rosae* (outgroup) | Tenthredinoidea: Tenthredinidae | GCF_917208135.1 | not applicable |
| *Bombus affinis* | Apoidea: Apidae | GCA_024516045.2 | not applicable |
| *Bombus ignitus* | Apoidea: Apidae | not applicable | DQ870926 |
| *Bombus vancouverensis* | Apoidea: Apidae | GCF_011952275.1 | not applicable |
| *Ceratina calcarata* | Apoidea: Apidae | GCF_001652005.2 | not applicable |
| *Chelonus insularis* | Ichneumonoidea: Braconidae | GCF_013357705.1 | not applicable |
| *Colletes gigas* | Apoidea: Colletidae | GCF_013123115.1 | not applicable |
| *Dufourea novaeangliae* | Apoidea: Halictidae | GCF_001272555.1 | not applicable |
| *Eufriesea mexicana* | Apoidea: Apidae | GCA_001483705.2 | not applicable |
| *Formica exsecta* | Formicoidea: Formicidae | GCF_003651465.1 | not applicable |
| *Frieseomelitta varia* | Apoidea: Apidae | GCF_011392965.1 | CM022150 |
| *Lepidotrigona flavibasis* | Apoidea: Apidae | not applicable | MN747147 |
| *Megachile rotundata* | Apoidea: Megachilidae | GCF_000220905.1 | not applicable |
| *Megalopta genalis* | Apoidea: Halictidae | GCA_011865705.1 | not applicable |
| *Melipona bicolor* | Apoidea: Apidae | GCA_030673865.1 | AF466146 |
| *Melipona quadrifasciata* | Apoidea: Apidae | GCA_001276565.1 | not applicable |
| *Nasonia vitripennis* | Chalcidoidea: Pteromalidae | GCF_009193385.2 | not applicable |
| *Nomada fabriciana* | Apoidea: Apidae | GCA_907165295.1 | not applicable |
| *Nomia melanderi* | Apoidea: Halictidae | GCF_003710045.2 | not applicable |
| *Osmia lignaria* | Apoidea: Megachilidae | GCF_012274295.1 | not applicable |
| *Solenopsis invicta* | Formicoidea: Formicidae | GCF_016802725.1 | not applicable |
| *Tetragonula pagdeni* | Apoidea: Apidae | not applicable | NC_066054 |
| *Trigonisca nataliae* | Apoidea: Apidae | not applicable | not yet available |
| *Vespa mandarinia* | Vespoidea: Vespidae | GCF_014083535.2 | not applicable |
