# Supplementary material for: The nuclear and mitochondrial genome assemblies of Tetragonisca angustula (Apidae: Meliponini), a tiny yet remarkable pollinator in the Neotropics
Source: BMC Genomics. 2024 Jun 11;25:587. doi: 10.1186/s12864-024-10502-z (PMC11167848; doi:10.1186/s12864-024-10502-z)
Supplement: Supplementary file 3 — Table S3. Contig Nx statistics, and other relevant metrics, of the de novo transcriptome of Tetragonisca angustula when all transcripts (2nd column) or only the longest isoforms (3rd column) are considered [file 12864_2024_10502_MOESM3_ESM.docx]

**Table S3** Contig Nx statistics, and other relevant metrics, of the *de novo* transcriptome of *Tetragonisca angustula* when all transcripts (2^nd^ column) or only the longest isoforms (3^rd^ column) are considered.

| Statistics | All transcript contigs (bp) | Longest isoforms only (bp) |
| --- | --- | --- |
| Contig N10 | 11,217 | 8,605 |
| Contig N20 | 8,246 | 5,770 |
| Contig N30 | 6,481 | 4,020 |
| Contig N40 | 5,140 | 2,757 |
| Contig N50 | 4,025 | 1,912 |
| Median contig length | 589 | 368 |
| Average contig | 1,603.9 | 918.5 |
| Total assembled bases | 221,884,685 | 85,459,584 |
