# Supplementary material for: The nuclear and mitochondrial genome assemblies of Tetragonisca angustula (Apidae: Meliponini), a tiny yet remarkable pollinator in the Neotropics
Source: BMC Genomics. 2024 Jun 11;25:587. doi: 10.1186/s12864-024-10502-z (PMC11167848; doi:10.1186/s12864-024-10502-z)
Supplement: Supplementary file 4 — Table S4. Estimations of the total size, and repetitive and unique lengths of the genome of Tetragonisca angustula based on a GenomeScope assessment [file 12864_2024_10502_MOESM4_ESM.docx]

**Table S4** Estimations of the total size, and repetitive and unique lengths of the genome of *Tetragonisca angustula* based on a GenomeScope assessment.

| Property | Minimum | Maximum |
| --- | --- | --- |
| Heterozygosity rate | 0.22% | 0.23% |
| Genome haploid length | 317,607,379 bp | 317,928,398 bp |
| Genome repeat length | 61,596,858 bp | 61,659,117 bp |
| Genome unique length | 256,010,521 bp | 256,269,282 bp |
| Model fit | 97.09% | 99.55% |
| Read error rate | 0.27% | 0.27% |
