# Supplementary material for: The nuclear and mitochondrial genome assemblies of Tetragonisca angustula (Apidae: Meliponini), a tiny yet remarkable pollinator in the Neotropics
Source: BMC Genomics. 2024 Jun 11;25:587. doi: 10.1186/s12864-024-10502-z (PMC11167848; doi:10.1186/s12864-024-10502-z)
Supplement: Supplementary file 5 — Table S5. Description of the repeat elements masked in the genome of Tetragonisca angustula for the purpose of gene prediction. 2nd column, total count; 3rd column, total number of bases masked; 4th column, relative size of the corresponding repeat element [file 12864_2024_10502_MOESM5_ESM.docx]

**Table S5** Description of the repeat elements masked in the genome of *Tetragonisca angustula* for the purpose of gene prediction. 2^nd^ column, total count; 3^rd^ column, total number of bases masked; 4^th^ column, relative size of the corresponding repeat element.

| Class | Count | bp masked | % masked |
| --- | --- | --- | --- |
| DNA | 810 | 199140 | 0.07% |
| CMC-EnSpm | 72 | 3860 | 0.00% |
| IS3EU | 79 | 22088 | 0.01% |
| Kolobok-Hydra | 2162 | 1005293 | 0.35% |
| Maverick | 335 | 82066 | 0.03% |
| Merlin | 1163 | 195604 | 0.07% |
| PiggyBac | 4351 | 809587 | 0.28% |
| Sola-1 | 428 | 114536 | 0.04% |
| TcMar-ISRm11 | 493 | 93536 | 0.03% |
| TcMar-Mariner | 5139 | 1274735 | 0.45% |
| TcMar-Tc1 | 24940 | 6425207 | 2.26% |
| TcMar-Tc4 | 3480 | 705549 | 0.25% |
| TcMar-Tigger | 981 | 172138 | 0.06% |
| hAT-Ac | 1222 | 394597 | 0.14% |
| hAT-Charlie | 2429 | 523603 | 0.18% |
| hAT-Pegasus | 60 | 14522 | 0.01% |
| hAT-Tip100 | 331 | 86837 | 0.03% |
| hAT-hAT19 | 86 | 55486 | 0.02% |
| LINE |  |  |  |
| CR1 | 1058 | 542442 | 0.19% |
| I | 2730 | 1920189 | 0.68% |
| I-Jockey | 1132 | 1318910 | 0.46% |
| L2 | 1259 | 293446 | 0.10% |
| R1 | 1497 | 1223188 | 0.43% |
| R2 | 65 | 29267 | 0.01% |
| R2-NeSL | 68 | 56600 | 0.02% |
| RTE-X | 658 | 533539 | 0.19% |
| LTR |  |  |  |
| Copia | 607 | 416626 | 0.15% |
| Gypsy | 1545 | 943574 | 0.33% |
| Pao | 168 | 192835 | 0.07% |
| Unknown | 1799 | 442662 | 0.16% |
| RC |  |  |  |
| Helitron | 923 | 3083641 | 1.08% |
| SINE |  |  |  |
| tRNA | 30 | 3421 | 0.00% |
| Unknown | 153430 | 28939886 | 10.18% |
| Total interspersed | 215530 | 52118610 | 18.32% |
| Low_complexity | 33402 | 1747054 | 0.61% |
| Simple_repeat | 196861 | 8974755 | 3.16% |
| TOTAL | **445793** | **62840419** | **22.09%** |
