# Supplementary material for: The nuclear and mitochondrial genome assemblies of Tetragonisca angustula (Apidae: Meliponini), a tiny yet remarkable pollinator in the Neotropics
Source: BMC Genomics. 2024 Jun 11;25:587. doi: 10.1186/s12864-024-10502-z (PMC11167848; doi:10.1186/s12864-024-10502-z)
Supplement: Supplementary file 6 — Table S6. Databases against which our gene annotation analyses were conducted. The numbers under “Matches” correspond to those of predicted protein-coding gene sequences that aligned against the corresponding database [file 12864_2024_10502_MOESM6_ESM.docx]

**Table S6** Databases against which our gene annotation analyses were conducted. The numbers under “Matches” correspond to those of predicted protein-coding gene sequences that aligned against the corresponding database.

| Analysis | Database | Matches |
| --- | --- | --- |
| BlastX |  |  |
|  | Swiss-Prot | 10,913 |
|  | TrEMBL | 5,119 |
|  | **Total** | 16,541 |
| InterProScan |  |  |
|  | PANTHER | 13,999 |
|  | Pfam | 13,442 |
|  | Reactome | 12,784 |
|  | Gene3D | 11,563 |
|  | GO | 10,973 |
|  | SUPERFAMILY | 10,930 |
|  | MetaCyc | 10,191 |
|  | MobiDBLite | 8,659 |
|  | ProSiteProfiles | 7,697 |
|  | SMART | 7,163 |
|  | CDD | 6,916 |
|  | Coils | 5,048 |
|  | ProSitePatterns | 4,022 |
|  | PRINTS | 3,123 |
|  | PIRSF | 986 |
|  | Hamap | 405 |
|  | SFLD | 106 |
|  | **Total** | 128,007 |
