# Supplementary material for: The nuclear and mitochondrial genome assemblies of Tetragonisca angustula (Apidae: Meliponini), a tiny yet remarkable pollinator in the Neotropics
Source: BMC Genomics. 2024 Jun 11;25:587. doi: 10.1186/s12864-024-10502-z (PMC11167848; doi:10.1186/s12864-024-10502-z)
Supplement: Supplementary file 9 — Table S9. Contaminants (bacteria) found among the raw genome sequencing data of Tetragonisca angustula [file 12864_2024_10502_MOESM9_ESM.docx]

**Table S9.** Contaminants (bacteria) found among the raw genome sequencing data of *Tetragonisca angustula*.

| Species | Group | Gram |
| --- | --- | --- |
| *Enterobacter ludwigii* | Enterobacteria | Negative |
| *Escherichia coli* | Enterobacteria | Negative |
| *Gilliamella apis* | Apibacteria | Positive |
| *Lactobacillus* sp. | Lactobacillus | Positive |
| *Neisseria* sp. | Neisseria | Negative |
| *Pantoea agglomerans* | Enterobacteria | Negative |
| *Proteus mirabilis* | Enterobacteria | Negative |
| *Pseudomonas* sp. | Pseudomonas | Negative |
| *Snodgrassella* sp. | Apibacteria | Positive |
| *Zophobihabitans entericus* | Enterobacteria | Negative |
