# Supplementary material for: The nuclear and mitochondrial genome assemblies of Tetragonisca angustula (Apidae: Meliponini), a tiny yet remarkable pollinator in the Neotropics
Source: BMC Genomics. 2024 Jun 11;25:587. doi: 10.1186/s12864-024-10502-z (PMC11167848; doi:10.1186/s12864-024-10502-z)
Supplement: Supplementary file 10 — Table S10. Orthogroup identification overview. 2nd column, number of orthologs identified by Orthofinder; 3rd column, number (and percentage) of identified orthologs that were assigned to orthogroups; 4th column, number (and percentage) of the identified orthologs that could not be assigned to any orthogroup; 5th column, number (and percentage) of orthogroups that were found in the genome of the corresponding species; 6th column, number of orthogroups that were found exclusively in the genome of the corresponding species; 7th column, number (and percentage) of the identified orthologs that were assigned to the orthogroups found exclusively in the genome of the corresponding species [file 12864_2024_10502_MOESM10_ESM.docx]

**Table S10** Orthogroup identification overview. 2^nd^ column, number of orthologs identified by Orthofinder; 3^rd^ column, number (and percentage) of identified orthologs that were assigned to orthogroups; 4^th^ column, number (and percentage) of the identified orthologs that could not be assigned to any orthogroup; 5^th^ column, number (and percentage) of orthogroups that were found in the genome of the corresponding species; 6^th^ column, number of orthogroups that were found exclusively in the genome of the corresponding species; 7^th^ column, number (and percentage) of the identified orthologs that were assigned to the orthogroups found exclusively in the genome of the corresponding species.

| Species | Orthologs | Orthologs in  orthogroups | Unassigned  orthologs | Orthogroups  containing species | Species-specific  orthogroups | Orthologs in species-specific orthogroups |
| --- | --- | --- | --- | --- | --- | --- |
| *Ampulex compressa* | 21,627 | 14,244 (65.9%) | 7,383 (34.1%) | 10,109 (47.1%) | 189 | 620 (2.9%) |
| *Andrena dorsata* | 20,625 | 19,873 (96.4%) | 752 (3.6%) | 10,172 (47.4%) | 50 | 115 (0.6%) |
| *Andrena hattorfiana* | 22,236 | 21,335 (95.9%) | 901 (4.1%) | 10,332 (48.1%) | 75 | 162 (0.7%) |
| *Apis laboriosa* | 20,470 | 20,421 (99.8%) | 49 (0.2%) | 9,394 (43.8%) | 7 | 23 (0.1%) |
| *Apis mellifera* | 23,471 | 23,360 (99.5%) | 111 (0.5%) | 9,473 (44.1%) | 34 | 102 (0.4%) |
| *Athalia rosae* | 26,342 | 25,882 (98.3%) | 460 (1.7%) | 9,988 (46.5%) | 457 | 1,833 (7.0%) |
| *Bombus affinis* | 28,024 | 27,941 (99.7%) | 83 (0.3%) | 9,565 (44.6%) | 56 | 757 (2.7%) |
| *Bombus vancouverensis* | 24,285 | 24,155 (99.5%) | 130 (0.5%) | 9,666 (45.0%) | 45 | 307 (1.3%) |
| *Ceratina calcarata* | 23,251 | 23,008 (99.0%) | 243 (1.0%) | 9,655 (45.0%) | 90 | 305 (1.3%) |
| *Chelonus insularis* | 19,220 | 18,914 (98.4%) | 306 (1.6%) | 9,106 (42.4%) | 248 | 822 (4.3%) |
| *Colletes gigas* | 18,601 | 18,533 (99.6%) | 68 (0.4%) | 9,324 (43.4%) | 29 | 180 (1.0%) |
| *Dufourea novaeangliae* | 12,157 | 12,119 (99.7%) | 38 (0.3%) | 9,221 (43.0%) | 4 | 11 (0.1%) |
| *Eufriesea mexicana* | 15,640 | 15,512 (99.2%) | 128 (0.8%) | 9,416 (43.9%) | 26 | 74 (0.5%) |
| *Formica exsecta* | 22,509 | 22,249 (98.8%) | 260 (1.2%) | 9,768 (45.5%) | 167 | 820 (3.6%) |
| *Frieseomelitta varia* | 23,628 | 23,485 (99.4%) | 143 (0.6%) | 9,834 (45.8%) | 36 | 126 (0.5%) |
| *Megachile rotundata* | 26,024 | 25,742 (98.9%) | 282 (1.1%) | 9,688 (45.1%) | 124 | 449 (1.7%) |
| *Megalopta genalis* | 22,381 | 22,228 (99.3%) | 153 (0.7%) | 9,257 (43.1%) | 59 | 213 (1.0%) |
| *Melipona bicolor* | 21,371 | 18,389 (86.0%) | 2,982 (14.0%) | 14,781 (68.9%) | 58 | 202 (0.9%) |
| *Melipona quadrifasciata* | 22,688 | 18,874 (83.2%) | 3,814 (16.8%) | 14,864 (69.3%) | 67 | 412 (1.8%) |
| *Nasonia vitripennis* | 34,173 | 33,435 (97.8%) | 738 (2.2%) | 9,985 (46.5%) | 979 | 5,060 (14.8%) |
| *Nomada fabriciana* | 15,301 | 14,825 (96.9%) | 476 (3.1%) | 9,231 (43.0%) | 45 | 99 (0.6%) |
| *Nomia melanderi* | 25,323 | 25,224 (99.6%) | 99 (0.4%) | 9,360 (43.6%) | 36 | 115 (0.5%) |
| *Osmia lignaria* | 25,907 | 25,787 (99.5%) | 120 (0.5%) | 9,592 (44.7%) | 78 | 323 (1.2%) |
| *Solenopsis invicta* | 30,910 | 30,486 (98.6%) | 424 (1.4%) | 10,201 (47.5%) | 435 | 2,460 (8.0%) |
| *Tetragonisca angustula* | 17,519 | 16,712 (95.4%) | 807 (4.6%) | 10,193 (47.5%) | 26 | 62 (0.4%) |
| *Vespa mandarinia* | 27,198 | 27,000 (99.3%) | 198 (0.7%) | 9,337 (43.5%) | 110 | 547 (2.0%) |
| Overall | 590,881 | 569,733 (96.4%) | 21,148 (3.6%) | - | 3,530 | 16,199 (2.8%) |
