# Supplementary material for: The nuclear and mitochondrial genome assemblies of Tetragonisca angustula (Apidae: Meliponini), a tiny yet remarkable pollinator in the Neotropics
Source: BMC Genomics. 2024 Jun 11;25:587. doi: 10.1186/s12864-024-10502-z (PMC11167848; doi:10.1186/s12864-024-10502-z)
Supplement: Supplementary file 11 — Table S11. Overview of the predicted orthogroup changes,, expansions (2nd to 4th columns) and contractions (5th to 7th columns), for the genome of each insect species analyzed. Numbers between parenthesis in the 2nd and 5th columns indicate the number of orthogroups found to be rapidly expanding and contracting, respectively. Positive or negative numbers in the 9th column indicate an overall expansion or contraction, respectively [file 12864_2024_10502_MOESM11_ESM.docx]

**Table S11** Overview of the predicted orthogroup changes, expansions (2^nd^ to 4^th^ columns) and contractions (5^th^ to 7^th^ columns), for the genome of each insect species analyzed. Numbers between parenthesis in the 2^nd^ and 5^th^ columns indicate the number of orthogroups found to be rapidly expanding and contracting, respectively. Positive or negative numbers in the 9^th^ column indicate an overall expansion or contraction, respectively.

| Species | Expanded orthogroups | Orthologs gained | Orthologs/  expansion | Contracted orthogroups | Orthologs lost | Orthologs/  contraction | No change | Avg. Expansion |
| --- | --- | --- | --- | --- | --- | --- | --- | --- |
| *Ampulex compressa* | 798 (88) | 1494 | 1.87 | 11870 (471) | 13903 | 1.17 | 8794 | -0.578185 |
| *Andrena dorsata* | 1587 (501) | 1958 | 1.23 | 1502 (143) | 1681 | 1.12 | 18373 | 0.0129065 |
| *Andrena hattorfiana* | 2250 (788) | 2977 | 1.32 | 1121 (96) | 1238 | 1.1 | 18091 | 0.0810269 |
| *Apis laboriosa* | 1444 (946) | 2469 | 1.71 | 1339 (175) | 1597 | 1.19 | 18679 | 0.04063 |
| *Apis mellifera* | 2229 (1640) | 4734 | 2.12 | 790 (76) | 923 | 1.17 | 18443 | 0.17757 |
| *Athalia rosae* | 3467 (341) | 7490 | 2.16 | 12986 (39) | 13497 | 1.04 | 5009 | -0.27989 |
| *Bombus affinis* | 2464 (1701) | 6512 | 2.64 | 650 (57) | 741 | 1.14 | 18348 | 0.268894 |
| *Bombus vancouverensis* | 1254 (762) | 2907 | 2.32 | 808 (84) | 922 | 1.14 | 19400 | 0.0924891 |
| *Ceratina calcarata* | 3199 (858) | 6814 | 2.13 | 8076 (186) | 8414 | 1.04 | 10187 | -0.0745504 |
| *Chelonus insularis* | 1713 (227) | 3529 | 2.06 | 14215 (161) | 15333 | 1.08 | 5534 | -0.549995 |
| *Colletes gigas* | 1991 (367) | 3611 | 1.81 | 2505 (268) | 3005 | 1.2 | 16966 | 0.028236 |
| *Dufourea novaeangliae* | 401 (45) | 578 | 1.44 | 4197 (1043) | 6024 | 1.44 | 16864 | -0.253751 |
| *Eufriesea mexicana* | 1092 (190) | 1873 | 1.72 | 9470 (554) | 10578 | 1.12 | 10900 | -0.405601 |
| *Formica exsecta* | 2139 (537) | 4347 | 2.03 | 2556 (421) | 3419 | 1.34 | 16767 | 0.0432392 |
| *Frieseomelitta varia* | 3656 (3108) | 7831 | 2.14 | 1220 (923) | 1295 | 1.06 | 16586 | 0.304538 |
| *Megachile rotundata* | 2973 (1208) | 6697 | 2.25 | 1511 (253) | 2021 | 1.34 | 16978 | 0.217873 |
| *Megalopta genalis* | 2366 (769) | 5103 | 2.16 | 1524 (253) | 1963 | 1.29 | 17572 | 0.146305 |
| *Melipona bicolor* | 856 (695) | 1392 | 1.63 | 1616 (957) | 1863 | 1.15 | 18990 | -0.0219458 |
| *Melipona quadrifasciata* | 721 (585) | 1858 | 2.58 | 1657 (787) | 1844 | 1.11 | 19084 | 0.000652316 |
| *Nasonia vitripennis* | 4515 (1179) | 15348 | 3.4 | 12091 (87) | 12631 | 1.04 | 4856 | 0.126596 |
| *Nomada fabriciana* | 1538 (111) | 1873 | 1.22 | 10237 (423) | 11940 | 1.17 | 9687 | -0.469062 |
| *Nomada melanderi* | 2871 (1245) | 7798 | 2.72 | 1338 (188) | 1662 | 1.24 | 17253 | 0.285901 |
| *Osmia lignaria* | 2568 (1031) | 6242 | 2.43 | 1367 (112) | 1521 | 1.11 | 17527 | 0.21997 |
| *Solenopsis invicta* | 3282 (1207) | 10757 | 3.28 | 1415 (108) | 1592 | 1.13 | 16765 | 0.427034 |
| *Tetragonisca angustula* | 1166 (843) | 1721 | 1.48 | 1712 (642) | 1958 | 1.14 | 18584 | -0.0110428 |
| *Vespa mandarinia* | 3736 (1167) | 10014 | 2.68 | 11326 (62) | 11744 | 1.04 | 6400 | -0.0806076 |
