# Supplementary material for: The nuclear and mitochondrial genome assemblies of Tetragonisca angustula (Apidae: Meliponini), a tiny yet remarkable pollinator in the Neotropics
Source: BMC Genomics. 2024 Jun 11;25:587. doi: 10.1186/s12864-024-10502-z (PMC11167848; doi:10.1186/s12864-024-10502-z)
Supplement: Supplementary file 12 — Table S12. Expansions (+) and contractions (-) in rapidly evolving odorant receptor orthogroups within Meliponini. “0” indicates that the orthogroup did not change in size for the corresponding species or node. <3>, ancestor of Meliponini; <5>, ancestor of Tetragonisca angustula plus Frieseomelitta varia; <1>, ancestor of Melipona [file 12864_2024_10502_MOESM12_ESM.docx]

**Table S12** Expansions (+) and contractions (-) in rapidly evolving odorant receptor orthogroups within Meliponini. “0” indicates that the orthogroup did not change in size for the corresponding species or node. <3>, ancestor of Meliponini; <5>, ancestor of *Tetragonisca angustula* plus *Frieseomelitta varia*; <1>, ancestor of *Melipona*.

| Orthogroup | <3> | *Tetragonisca*  *angustula* | *Frieseomelitta*  *varia* | <5> | *Melipona*  *bicolor* | *Melipona*  *quadrifasciata* | <1> | Total |
| --- | --- | --- | --- | --- | --- | --- | --- | --- |
| OG0000021 | 0 | 0 | +2 | 0 | 0 | -5 | 0 | -3 |
| OG0000041 | 0 | -12 | 0 | 0 | +5 | 0 | 0 | -7 |
| OG0000062 | 0 | 0 | +5 | +7 | 0 | -3 | +9 | +18 |
| OG0000063 | 0 | 0 | +18 | 0 | -4 | 0 | 0 | +14 |
| OG0000066 | 0 | -8 | +3 | 0 | 0 | 0 | 0 | -5 |
| OG0000075 | 0 | +5 | -3 | 0 | +2 | -2 | 0 | +2 |
| OG0000112 | 0 | +2 | 0 | 0 | -4 | 0 | -3 | -5 |
| OG0000124 | 0 | 0 | +2 | 0 | +4 | 0 | 0 | +6 |
| OG0000143 | +4 | 0 | 0 | +6 | 0 | +4 | 0 | +14 |
| OG0000264 | -3 | 0 | 0 | 0 | 0 | 0 | 0 | -3 |
| OG0000326 | 0 | 0 | 0 | -1 | 0 | 0 | 0 | -1 |
| OG0000339 | +3 | 0 | 0 | 0 | +2 | 0 | 0 | +5 |
| OG0000379 | -2 | 0 | 0 | 0 | 0 | +1 | 0 | -1 |
| OG0001499 | 0 | 0 | +1 | 0 | +1 | 0 | 0 | +2 |
| OG0002257 | 0 | 0 | 0 | 0 | +2 | -2 | 0 | 0 |
| OG0006085 | 0 | 0 | 0 | 0 | 0 | 0 | +1 | +1 |
| OG0006475 | 0 | 0 | 0 | 0 | 0 | +1 | 0 | +1 |
| OG0010406 | 0 | -1 | +5 | 0 | -1 | +2 | 0 | +5 |
| OG0010578 | 0 | 0 | 0 | -1 | 0 | +6 | 0 | +5 |
| OG0012179 | 0 | 0 | -1 | 0 | 0 | 0 | -1 | -2 |
| OG0013687 | 0 | -1 | +1 | 0 | 0 | -1 | 0 | -1 |
| OG0013807 | 0 | 0 | 0 | -1 | +1 | -1 | 0 | -1 |
| OG0016720 | 0 | 0 | 0 | -1 | 0 | 0 | 0 | -1 |
| OG0020765 | 0 | 0 | 0 | -1 | -1 | +1 | 0 | -1 |
| TOTAL | +2 | -15 | +33 | +8 | +7 | +1 | +6 | +42 |
