# Supplementary material for: The nuclear and mitochondrial genome assemblies of Tetragonisca angustula (Apidae: Meliponini), a tiny yet remarkable pollinator in the Neotropics
Source: BMC Genomics. 2024 Jun 11;25:587. doi: 10.1186/s12864-024-10502-z (PMC11167848; doi:10.1186/s12864-024-10502-z)
Supplement: Supplementary file 13 — Table S13. Predicted number of orthogroups in expansion (2nd column) and contraction (3rd column) in for the genome of each sampled stingless bee species based on OrthoVenn analysis [file 12864_2024_10502_MOESM13_ESM.docx]

**Table S13** Predicted number of orthogroups in expansion (2^nd^ column) and contraction (3^rd^ column) for the genome of each sampled stingless bee species based on OrthoVenn analysis.

| Species | Expansion | Contraction |
| --- | --- | --- |
| *Frieseomelitta varia* | 3802 | 3121 |
| *Melipona bicolor* | 545 | 2232 |
| *Melipona quadrifasciata* | 578 | 1488 |
| *Tetragonisca angustula* | 583 | 2212 |
