# Supplementary material for: The nuclear and mitochondrial genome assemblies of Tetragonisca angustula (Apidae: Meliponini), a tiny yet remarkable pollinator in the Neotropics
Source: BMC Genomics. 2024 Jun 11;25:587. doi: 10.1186/s12864-024-10502-z (PMC11167848; doi:10.1186/s12864-024-10502-z)
Supplement: Supplementary file 14 — Table S14. Genome profile of the stingless bee species sequenced to date and the NCBI accessions for their data [file 12864_2024_10502_MOESM14_ESM.docx]

**Table S14** Genome profile of the stingless bee species sequenced to date and the NCBI accessions for their data.

| Species | Read  type | Genome  length (Mb) | Contig  count | Longest  contig (Mb) | N50  (kb) | L50 | NCBI  accession |
| --- | --- | --- | --- | --- | --- | --- | --- |
| *Frieseomelitta varia* | short | 275.42 | 2,173 | 2.25 | 467.00 | 176 | GCA_011392965.1 |
| *Heterotrigona itama* | short | 262.45 | 13,733 | 0.43 | 49.82 | 1,383 | GCA_903986555.1 |
| *Lepidotrigona ventralis* | short | 333.08 | 123,317 | 0.27 | 6.64 | 10,262 | GCA_002806875.1 |
| *Melipona bicolor* | long | 259.85 | 241 | 36.76 | 6,226.00 | 12 | GCA_030673865.1 |
| *Melipona quadrifasciata* | short | 256.30 | 2,866 | 12.08 | 1,860.00 | 42 | GCA_001276565.1 |
| *Tetragonula carbonaria* | short | 299.55 | 90,201 | 0.18 | 13.59 | 5,949 | GCA_010645115.1 |
| *Tetragonula clypearis* | short | 294.05 | 77,144 | 0.17 | 14.85 | 5,525 | GCA_010645135.1 |
| *Tetragonula davenporti* | short | 284.61 | 47,286 | 0.27 | 18.51 | 4,237 | GCA_010645165.1 |
| *Tetragonula hockingsi* | short | 307.10 | 112,753 | 0.20 | 10.50 | 7,605 | GCA_010645185.1 |
| *Tetragonula mellipes* | short | 337.57 | 133,257 | 0.24 | 16.39 | 5,285 | GCA_011634685.1 |
