# Supplementary material for: The nuclear and mitochondrial genome assemblies of Tetragonisca angustula (Apidae: Meliponini), a tiny yet remarkable pollinator in the Neotropics
Source: BMC Genomics. 2024 Jun 11;25:587. doi: 10.1186/s12864-024-10502-z (PMC11167848; doi:10.1186/s12864-024-10502-z)

Fig. S1 Six quality parameters of the PacBio long-read sequencing obtained through a LongQC assessment.

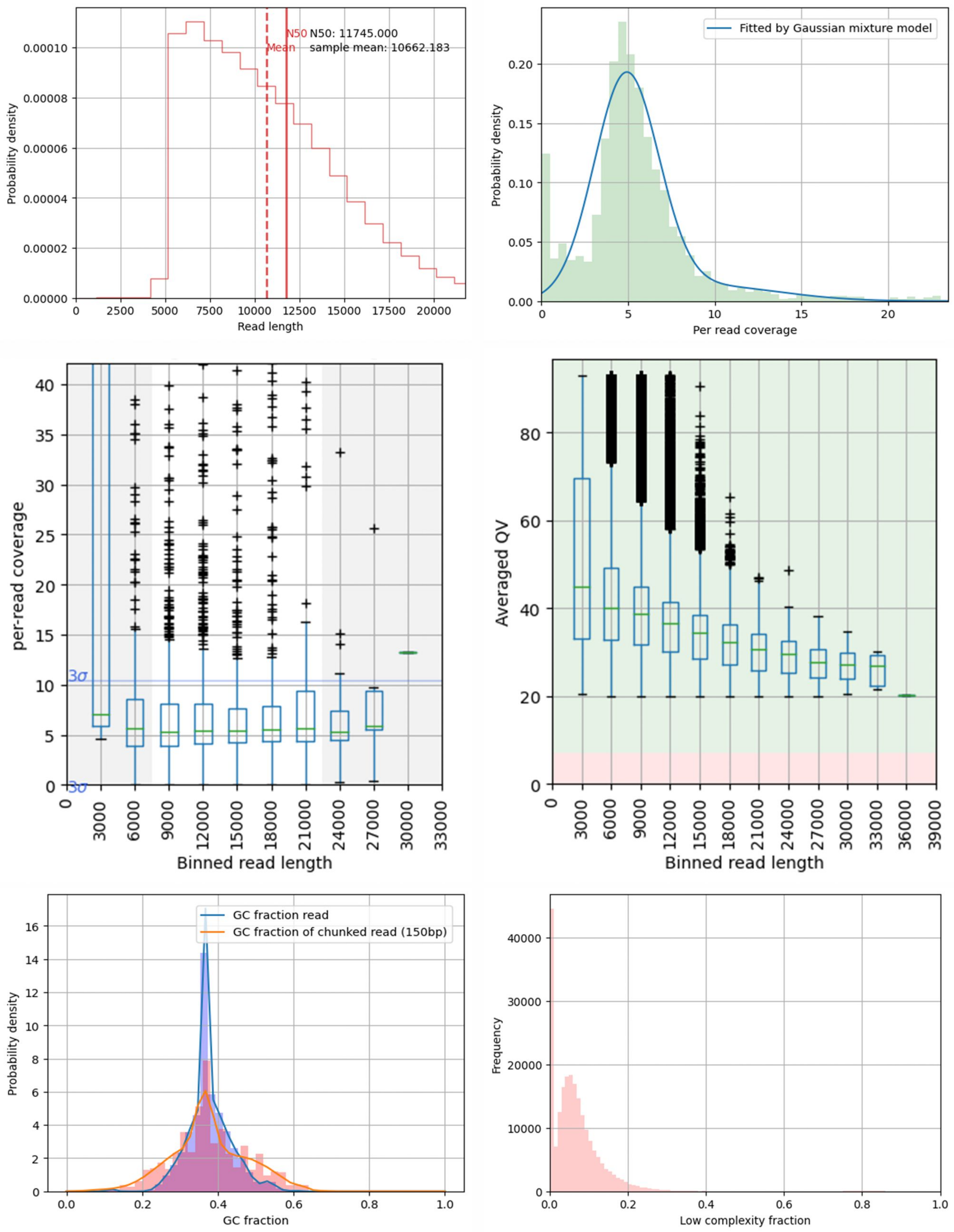

Supplement: Supplementary file 15 — Fig. S1. Six quality parameters of the PacBio long-read sequencing obtained through a LongQC assessment [file 12864_2024_10502_MOESM15_ESM.pdf]
