# Supplementary figures and images for: The nuclear and mitochondrial genome assemblies of Tetragonisca angustula (Apidae: Meliponini), a tiny yet remarkable pollinator in the Neotropics
Source: BMC Genomics. 2024 Jun 11;25:587. doi: 10.1186/s12864-024-10502-z (PMC11167848; doi:10.1186/s12864-024-10502-z)

Fig. S2 Eight quality parameters of 2×101 (SR1) Illumina short-read sequencing (R1) based on FastQC analysis.

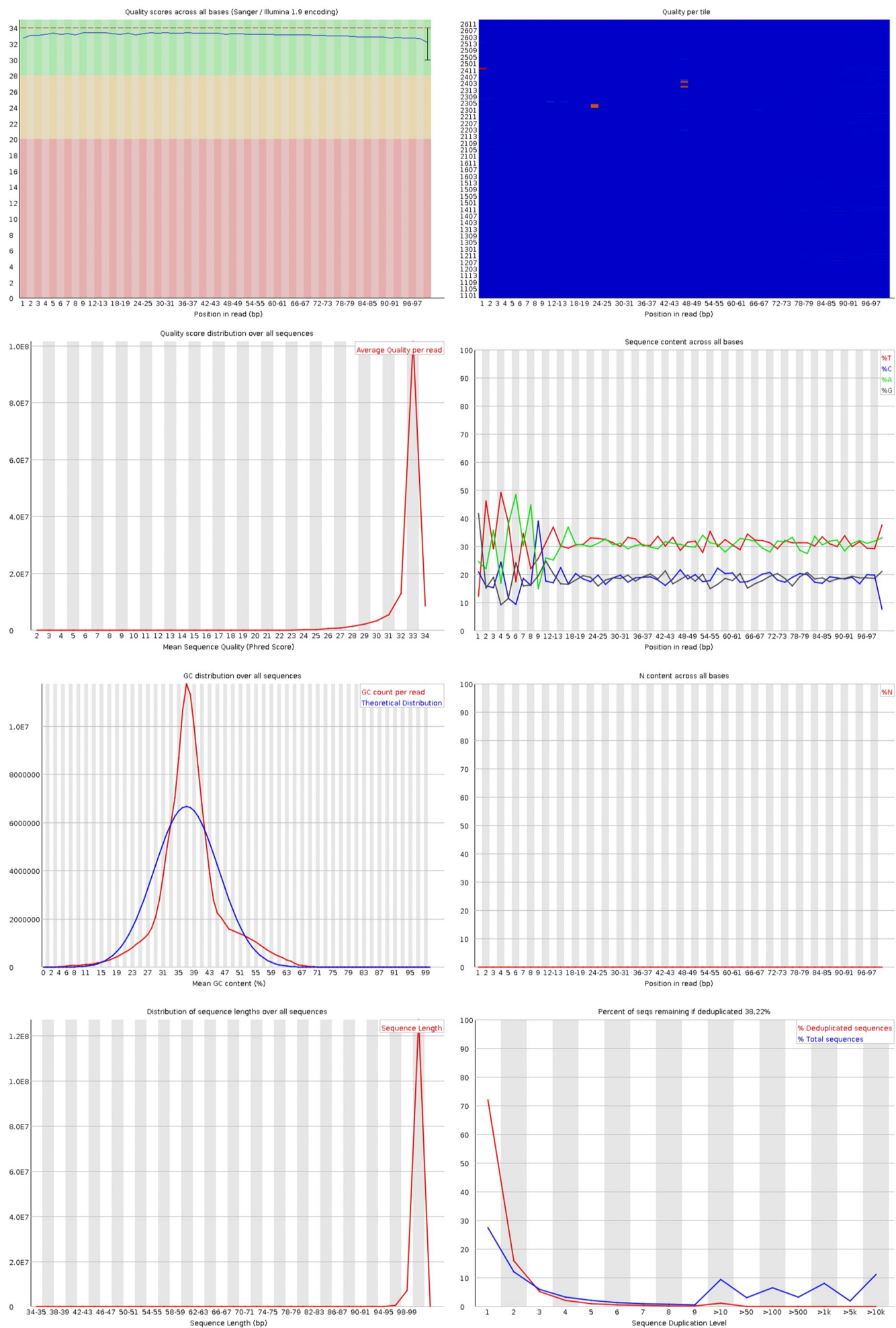

Supplement: Supplementary file 16 — Fig. S2. Eight quality parameters of 2 × 101 (SR1) Illumina short-read sequencing (R1) based on FastQC analysis [file 12864_2024_10502_MOESM16_ESM.pdf]

Fig. S3 Eight quality parameters of 2×101 (SR1) Illumina short-read sequencing (R2) based on FastQC analysis.

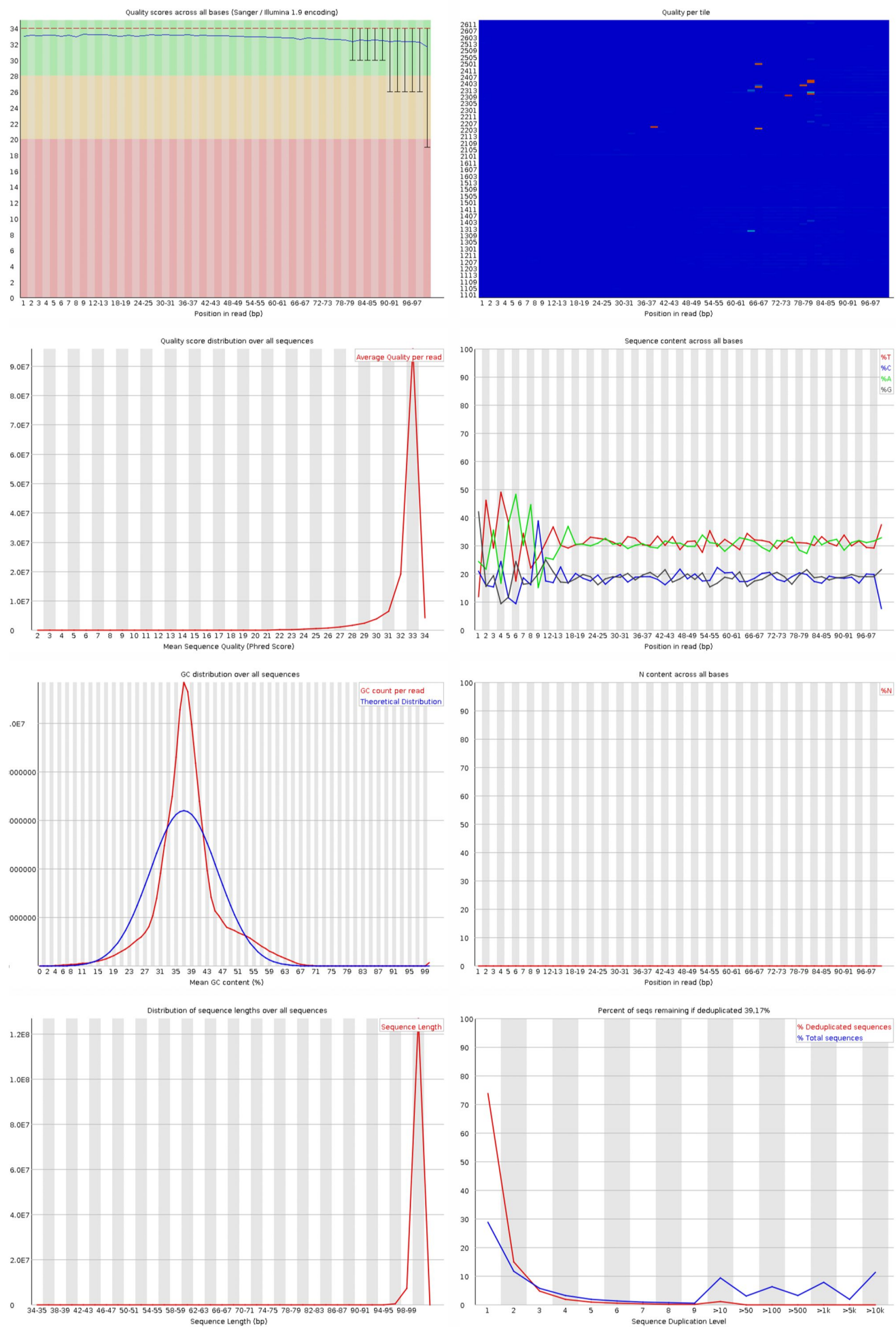

Supplement: Supplementary file 17 — Fig. S3. Eight quality parameters of 2 × 101 (SR1) Illumina short-read sequencing (R2) based on FastQC analysis [file 12864_2024_10502_MOESM17_ESM.pdf]

Fig. S5 Eight quality parameters of 2×301 (SR3) Illumina short-read sequencing (R2) based on FastQC analysis.

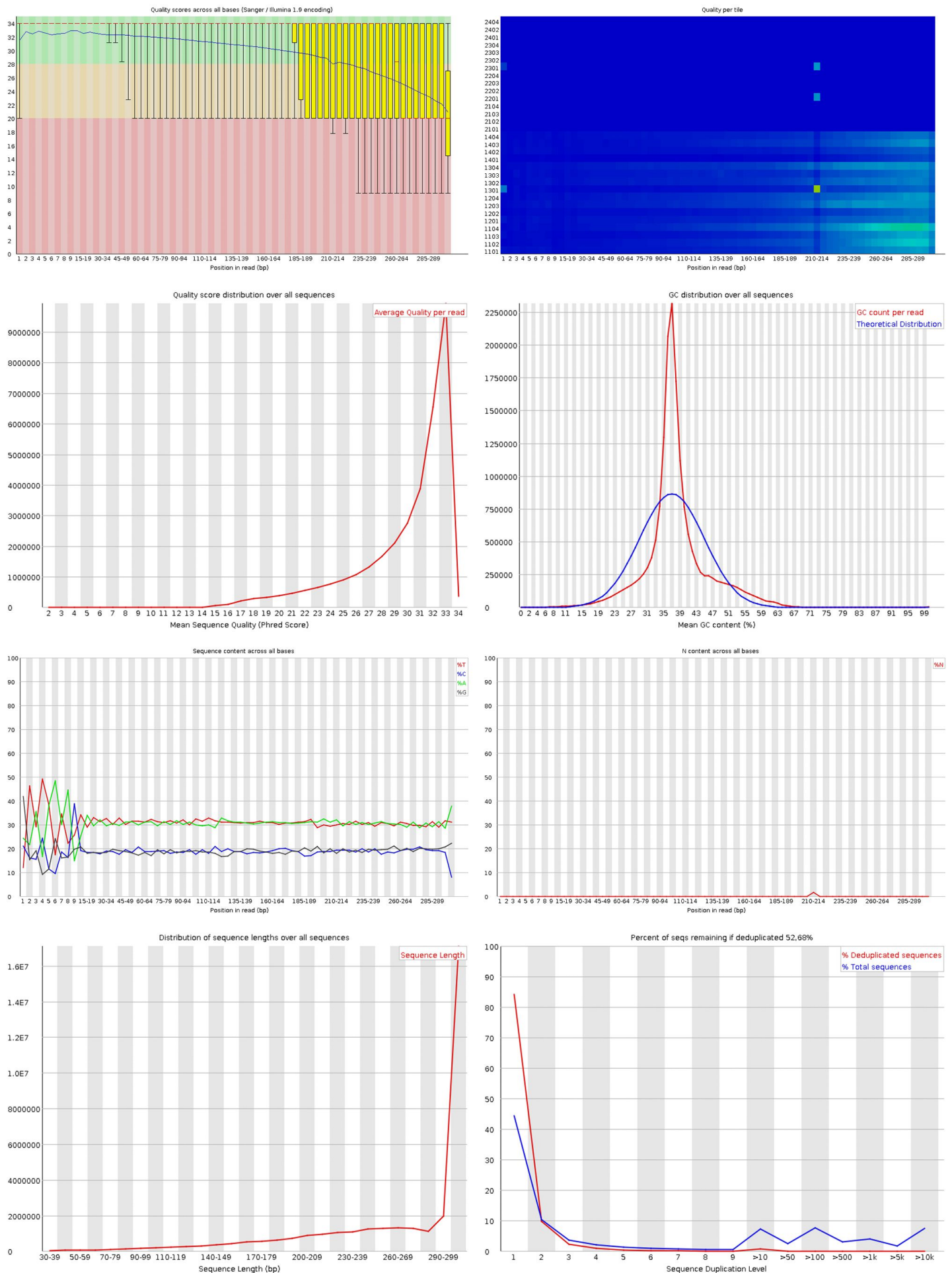

Supplement: Supplementary file 19 — Fig. S5. Eight quality parameters of 2 × 301 (SR3) Illumina short-read sequencing (R2) based on FastQC analysis [file 12864_2024_10502_MOESM19_ESM.pdf]
