# Supplementary material for: The nuclear and mitochondrial genome assemblies of Tetragonisca angustula (Apidae: Meliponini), a tiny yet remarkable pollinator in the Neotropics
Source: BMC Genomics. 2024 Jun 11;25:587. doi: 10.1186/s12864-024-10502-z (PMC11167848; doi:10.1186/s12864-024-10502-z)

Fig. S4 Eight quality parameters of 2×301 (SR3) Illumina short-read sequencing (R1) based on FastQC analysis.

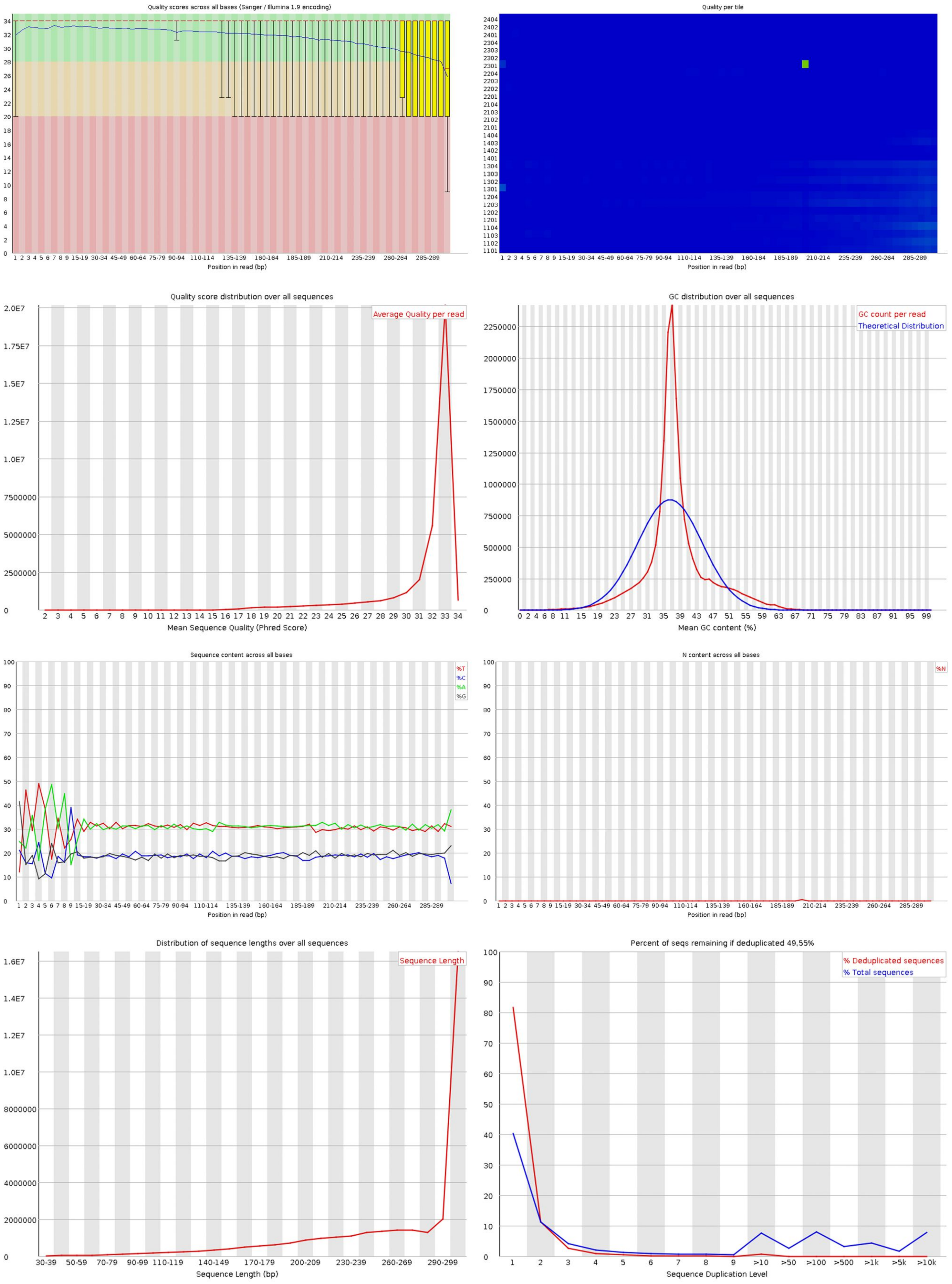

Supplement: Supplementary file 18 — Fig. S4. Eight quality parameters of 2 × 301 (SR3) Illumina short-read sequencing (R1) based on FastQC analysis [file 12864_2024_10502_MOESM18_ESM.pdf]
