# Supplementary material for: The nuclear and mitochondrial genome assemblies of Tetragonisca angustula (Apidae: Meliponini), a tiny yet remarkable pollinator in the Neotropics
Source: BMC Genomics. 2024 Jun 11;25:587. doi: 10.1186/s12864-024-10502-z (PMC11167848; doi:10.1186/s12864-024-10502-z)

Fig. S6 Contig ExN50 plot of the de novo-assembled transcriptome of *Tetragonisca angustula* showing a peak at E95 of roughly 4.7 kb.

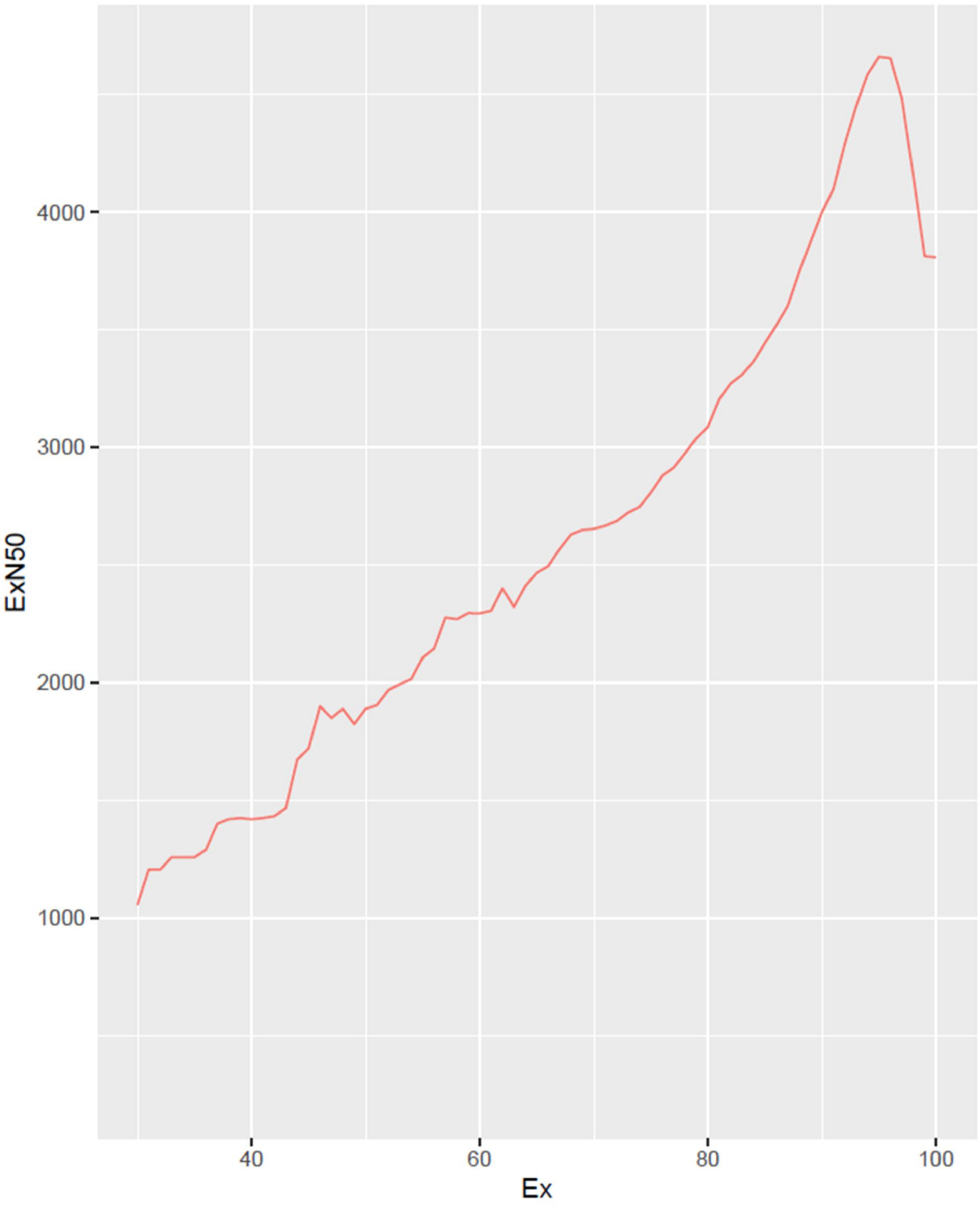

Supplement: Supplementary file 20 — Fig. S6. Contig ExN50 plot of the de novo-assembled transcriptome of Tetragonisca angustula showing a peak at E95 of roughly 4.7 kb [file 12864_2024_10502_MOESM20_ESM.pdf]
