# Supplementary material for: The nuclear and mitochondrial genome assemblies of Tetragonisca angustula (Apidae: Meliponini), a tiny yet remarkable pollinator in the Neotropics
Source: BMC Genomics. 2024 Jun 11;25:587. doi: 10.1186/s12864-024-10502-z (PMC11167848; doi:10.1186/s12864-024-10502-z)

Fig. S7 GenomeScope profile plots of absolute (top graph) and log-transformed (bottom graph) k-mer frequency distributions, at a k-mer length of 21, built based on the SR1 reads.

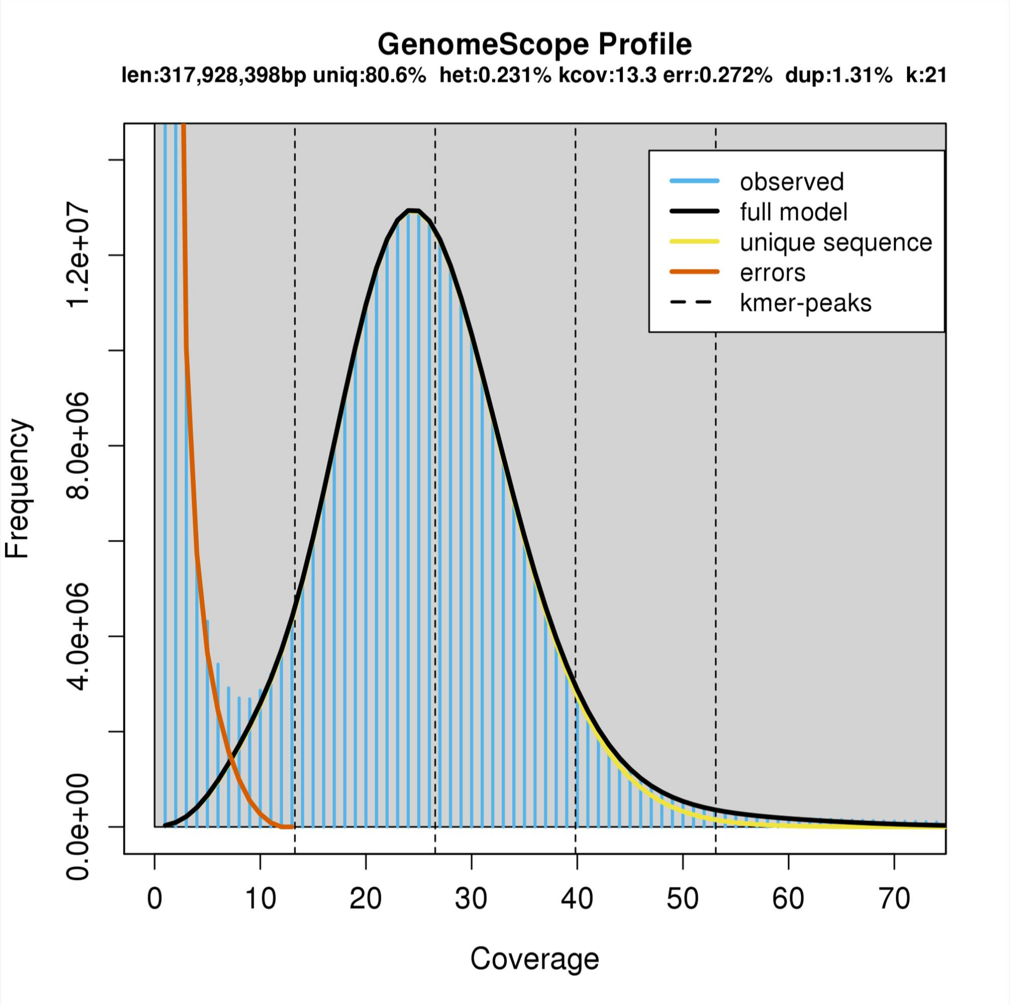

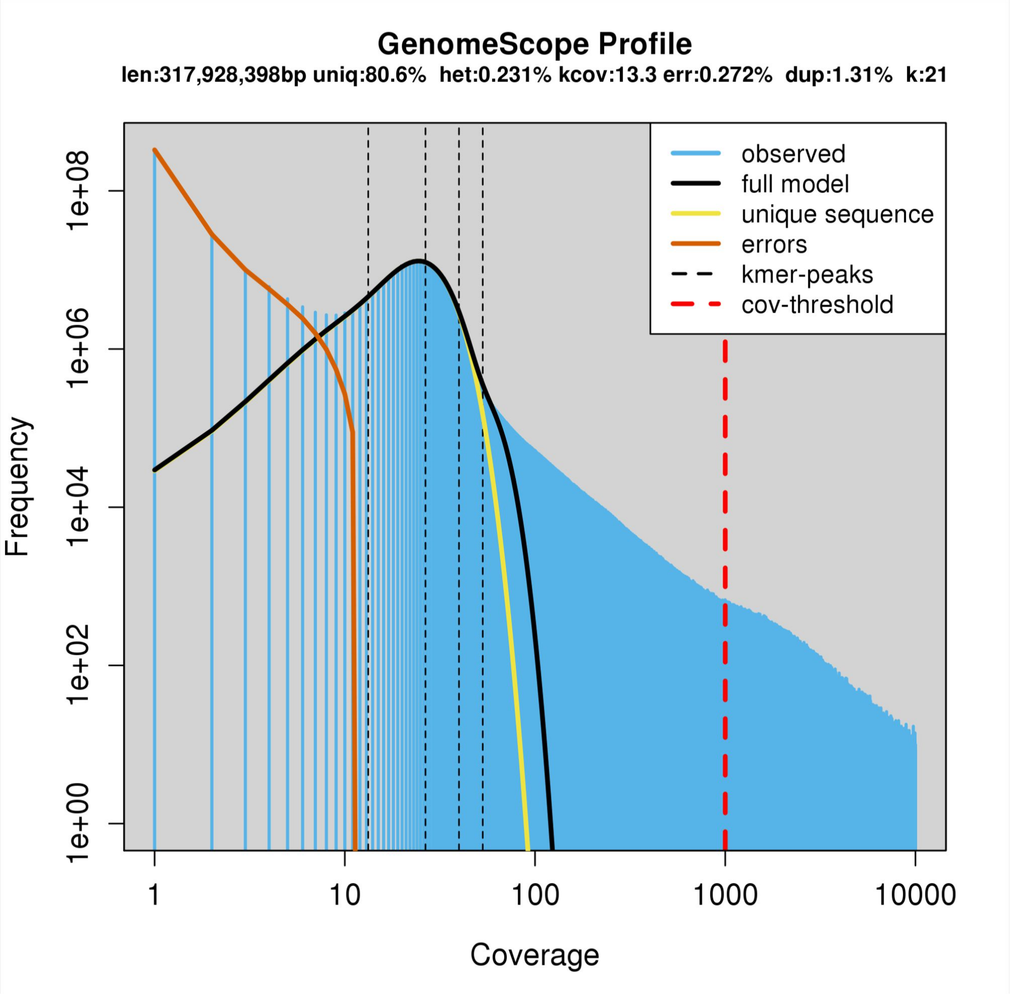

Supplement: Supplementary file 21 — Fig. S7. GenomeScope profile plots of absolute (top graph) and log-transformed (bottom graph) k-mer frequency distributions, at a k-mer length of 21, built based on the SR1 reads [file 12864_2024_10502_MOESM21_ESM.pdf]
